# Predictive design of tissue-specific mammalian enhancers that function in the mouse embryo

**DOI:** 10.1038/s41588-026-02729-1

**Published:** 2026-08-25

**Authors:** Shenzhi Chen, Vincent Loubiere, Ethan W. Hollingsworth, Ken Murakami, Nikolaus Mandlburger, Sandra H. Jacinto, Atrin Dizehchi, Jacob Schreiber, Evgeny Z. Kvon, Alexander Stark

**Affiliations:** 1 Research Institute of Molecular Pathology (IMP), Vienna BioCenter (VBC), Vienna, Austria; 2 Vienna BioCenter PhD Program, Doctoral School of the University of Vienna and Medical University of Vienna, Vienna, Austria; 3 Department of Developmental and Cell Biology, University of California Irvine, Irvine, CA USA; 4 Medical Scientist Training Program, University of California Irvine, Irvine, CA USA; 5 Department of Systems Biology, University of California Irvine, Irvine, CA USA; 6 Medical University of Vienna, Vienna BioCenter (VBC), Vienna, Austria; 7 Present Address: Department of Genomics and Computational Biology, UMass Chan Medical School, Worcester, MA USA

**Keywords:** Computational biology and bioinformatics, Synthetic biology, Gene regulation, Functional genomics

## Abstract

Enhancers control tissue-specific gene expression across animals^1^. Although deep learning^2,3^ has enabled enhancer prediction and design in mammalian cell lines and non-mammalian model organisms^4–10^ (reviewed in a previous publication^11^), it remains unclear whether such approaches can operate within the regulatory complexity of mammalian genomes and tissues in vivo. Here we present a general strategy for designing tissue-specific enhancers that function reliably in mice. We use deep learning to train compact convolutional neural networks on curated chromatin accessibility data and fine-tune them by transfer learning on validated human and mouse enhancers. Guided by these models, we design 15 synthetic enhancers for the heart, limb and central nervous system in mouse embryos, all of which are active in their intended target tissue. These results demonstrate that mammalian enhancer function can be reliably inferred from DNA sequence alone, enabling the predictive de novo design of tissue-specific synthetic enhancers from modest training sets. This work establishes a generalizable framework for programmable control of mammalian gene expression in vivo, opening new avenues in functional genomics, synthetic biology and gene therapy.

## Abstract (web summary)

Researchers used AI to design synthetic transcriptional enhancers that are active in specific tissues in mammals and validated their function in mice. This type of approach could enable advanced gene expression control in synthetic biology and gene therapy.

## Main

**Fig. 1:**
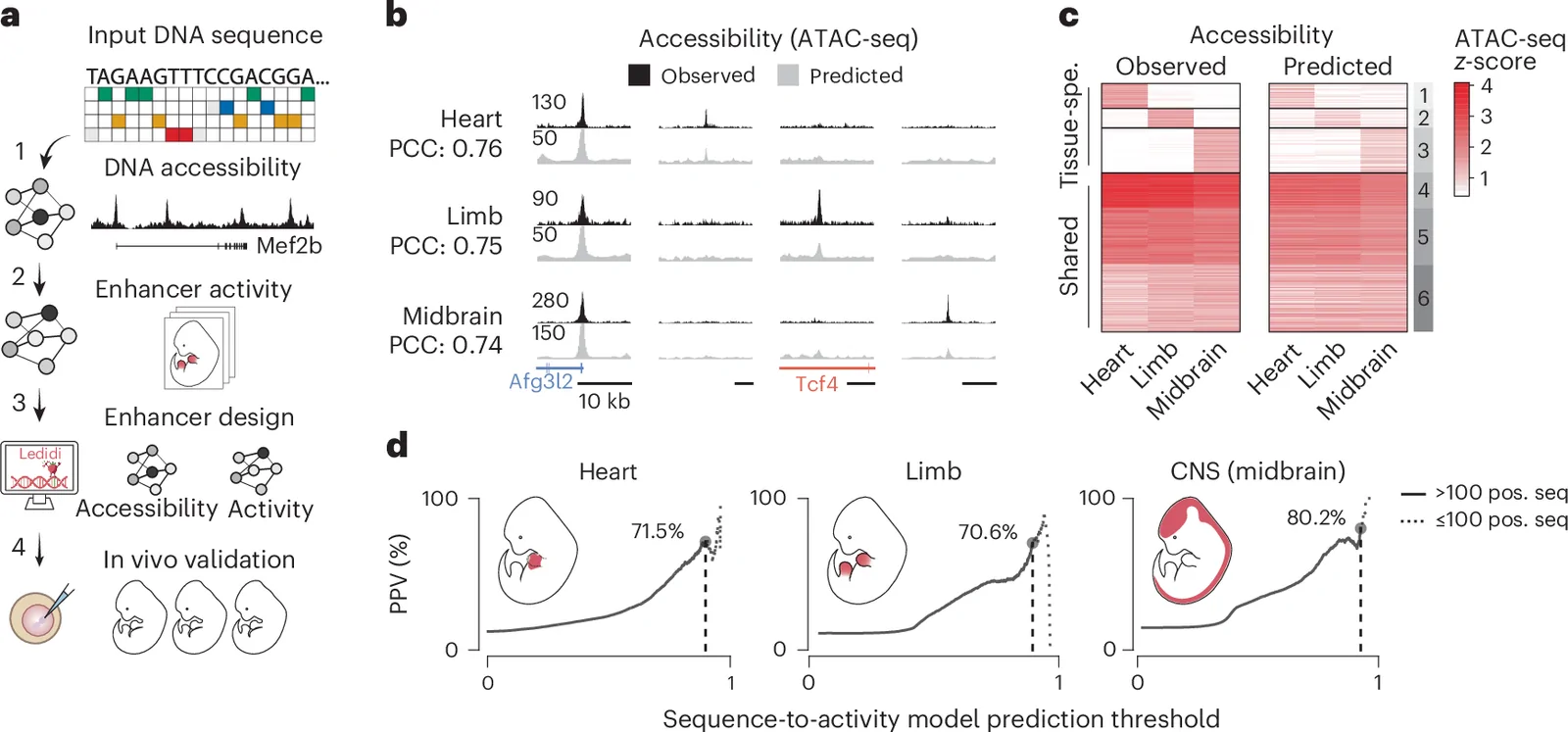
Deep learning-based prediction and design of tissue-specific mammalian enhancers. **a**, Schematic overview of the deep learning and transfer learning approach to enhancer design in mammals. (1) Deep learning models are pre-trained on genome-wide DNA accessibility data before being (2) fine-tuned using transfer learning on a subset of tissue-specific enhancers from the VISTA Enhancer Browser^13^. (3) Both models are used to guide the design of synthetic enhancers using Ledidi^22^ before (4) assessing their activity and specificity in E11.5 mouse embryos using reporter assays^23^. **b**, Screenshot of experimentally observed (black) and predicted (gray) ATAC-seq signals at one shared and three tissue-specific ATAC-seq peaks on mouse chromosome 18 (coordinates: 67,440,557–67,458,528; 3,637,665–3,694,606; 69,599,055–69,635,656; 3,825,318–3,854,833), which was not used for training. Genes on the positive (orange) and negative (in blue) strands are shown on the bottom. **c**, Scaled experimentally observed (left) and predicted (right) ATAC-seq signals for three tissues (shades of red; see color bar) on held-out genomic regions that were not used for training. *K*-means clusters are shown on the right (*k* = 6). Number of sequences: *n* = 474, 368, 849, 666, 1,070 and 1,270. Clusters 4–6 are accessible in all three tissues. Spe., specific. **d**, PPV of enhancer activity predictions across prediction score thresholds. For each threshold (*x* axis, 0–1), the *y* axis shows the percentage of active enhancers (positives) among all predicted enhancers (predicted score ≥ threshold; number of sequences: *n* = 31,636). The maximum value is reported when at least 100 positive sequences (pos. seq) remain (dashed line).

We reasoned that integrating genome-wide chromatin accessibility data from fetal mouse tissues with functionally validated embryonic enhancer sequences through transfer learning might enable predictive enhancer design in mammals (Fig. 1a), similar to recent work in *Drosophila* embryos^5^.

**Extended Data Fig. 1:**
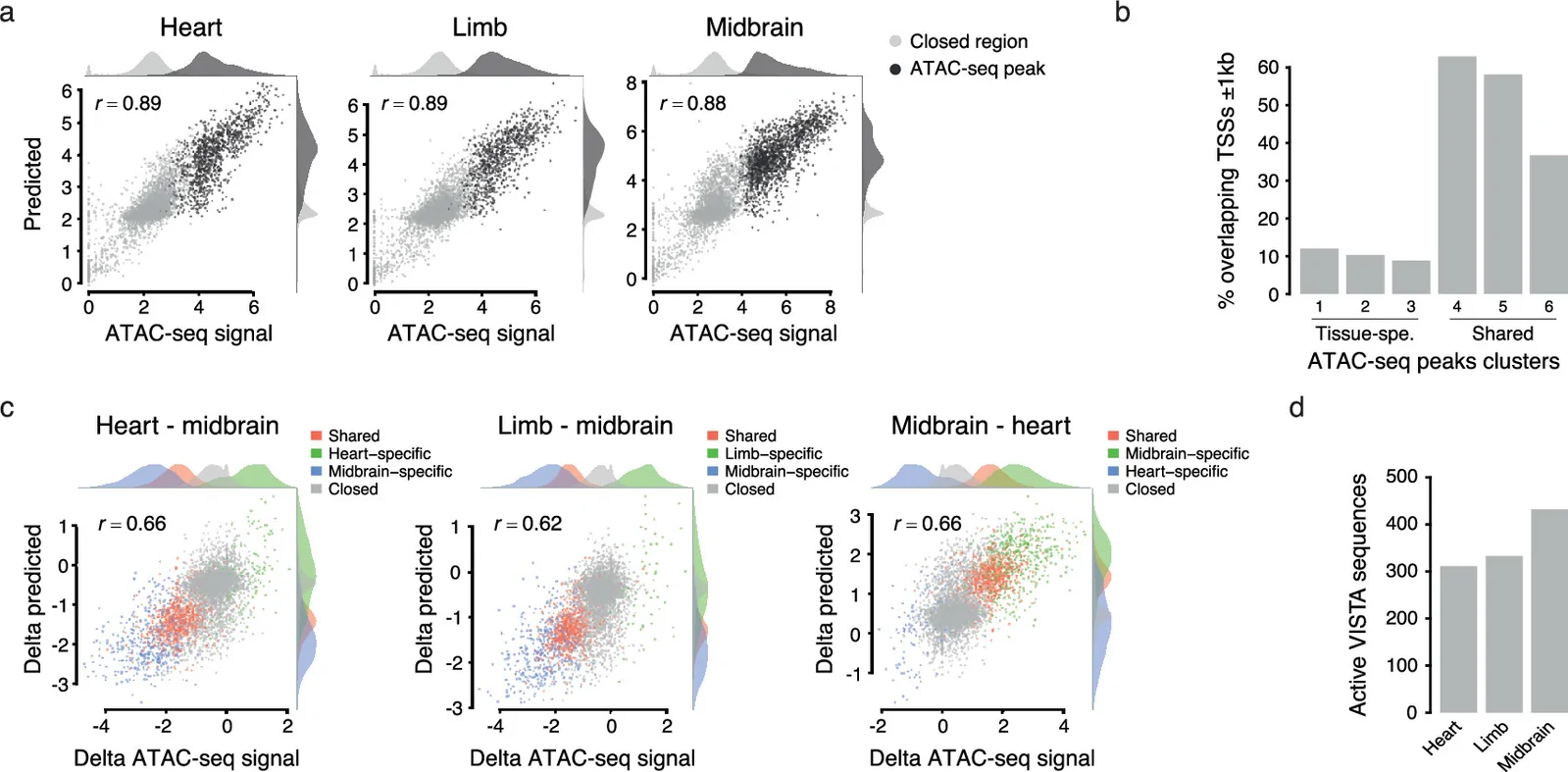
Performance of sequence-to-accessibility models and composition of training datasets. **a-** Observed vs. predicted ATAC-Seq signal on sequences that were not used for training (test set). Number of sequences n = 229,557; 222,813; 259,747 for heart, limb and midbrain, respectively. **b-** Percentage of ATAC-seq peaks overlapping Transcription Start Sites (TSSs) +/− 1 kb for the shared versus tissue-specific groups of peaks. **c-** Delta observed vs. predicted ATAC-Seq signal on sequences that were not used for training (test set), further stratified based on their tissue-specificity (color legend). Number of sequences *n* = 216,177. **d-** Number of annotated enhancers in the VISTA enhancer browser database for the indicated tissues.

We first trained sequence-to-accessibility models on published assay for transposase-accessible chromatin using sequencing (ATAC-seq) datasets^12^ for E11.5 mouse heart, limb and midbrain tissues, augmenting accessible regions while downsampling inaccessible regions to balance their respective contributions. These models accurately predicted DNA accessibility on genomic regions excluded from training (Pearson correlation coefficient (PCC) ≥ 0.76 on tiled chromosome 18 (Fig. 1b) and PCC ≥ 0.88 on test set sequences from all chromosomes, balanced like the training set (Extended Data Fig. 1a and Supplementary Information)) and captured both shared and tissue-specific ATAC-seq peaks (Fig. 1c and Extended Data Fig. 1b). Notably, pairwise comparisons of experimental and predicted data between different tissues demonstrated robust prediction of differential chromatin accessibility; that is, tissue-specific peaks (PCC ≥ 0.62; Extended Data Fig. 1c), making these models strong starting points to derive enhancer activity rules.

**Extended Data Fig. 2:**
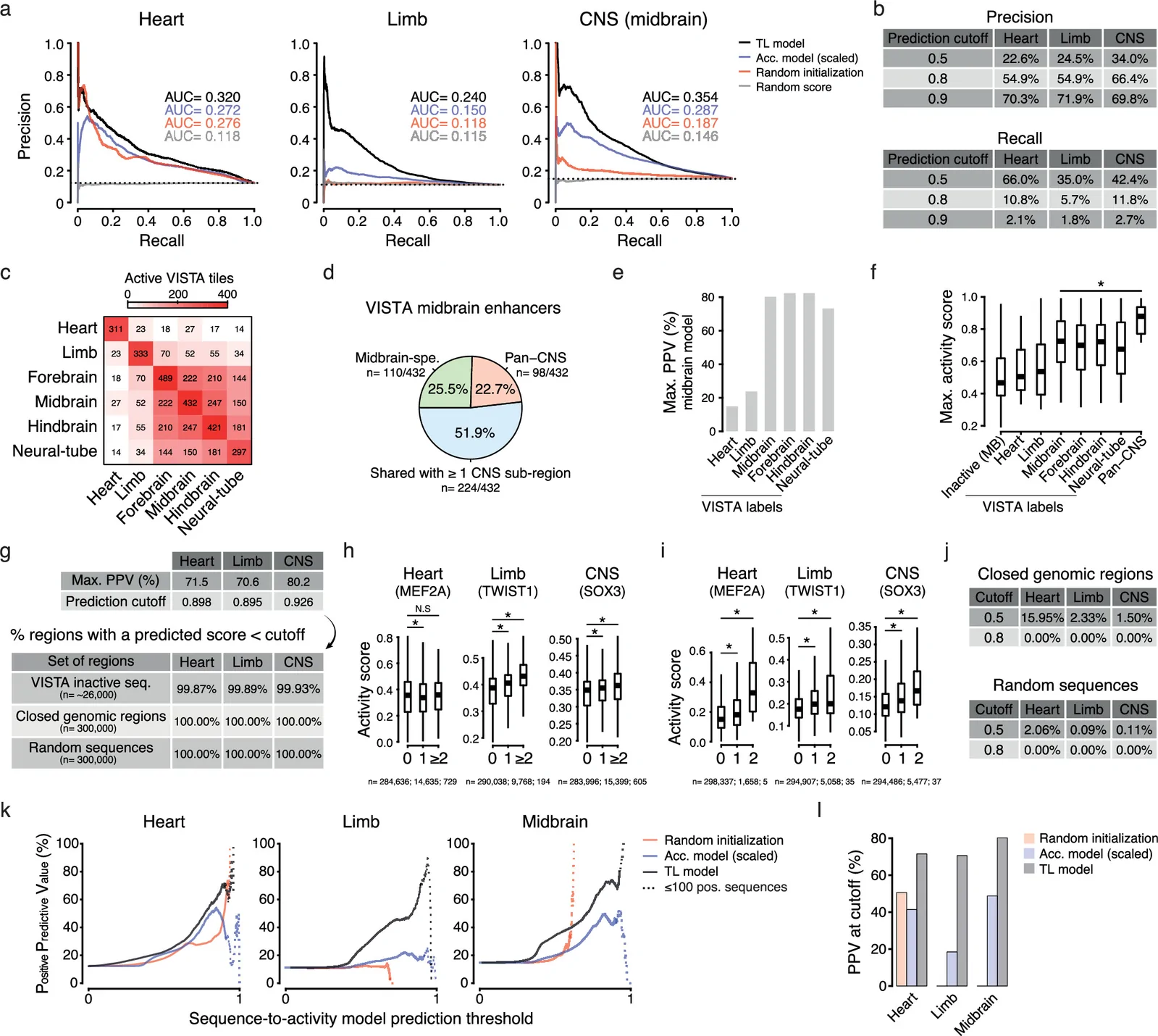
Performance of sequence-to-activity models and composition of training datasets. **a-** Precision-recall curves of sequence-to-activity models (black), scaled accessibility models (blue), randomly initialized models directly trained on VISTA sequences (red) and a random predictor (grey). **b-** Precision and recall by sequence-to-activity models at increasing activity score thresholds. **c-** Overlap between tissue labels from VISTA. **d-** Percentage of VISTA midbrain enhancers active in: only midbrain (green), at least one other CNS subregion (blue), all CNS subregions (Pan-CNS, red). **e-** Maximum Positive Predictive Value (%) of the midbrain sequence-to-activity model, evaluated on VISTA enhancers active in heart, limb, or any of the four CNS subregions (x-axis; maximum value at ≥100 positives. **f-** Midbrain-model activity score for VISTA sequences inactive in midbrain (MB), active in heart, limb, any of the four CNS subregions, or all CNS subregions (pan-CNS; x-axis; only max score per enhancer). Box plots show the median (center line), interquartile range (box), and whiskers extending to the most extreme data points within 1.5× the interquartile range (Tukey box plots), without outliers; two-sided Wilcoxon test: *P < 0.05. **g-** Top: maximum Positive Predictive Values (%) and corresponding threshold. Bottom: inactive predictions (%) for three different sets of held-out controls. **h-i-** Activity scores of 300,000 closed genomic regions (**h**) and 300,000 random sequences (**i**), stratified by the number of tissue-specific motifs they contain (top). Tukey box plots without outliers. Two-sided Wilcoxon test: *P < 0.05. **j-** Percentage of closed genomic regions and random sequences reaching increasing predicted activity thresholds. **k-** PPV of enhancer activity across prediction score thresholds, comparing a scaled sequence-to-accessibility model (blue) and a randomly initialized model trained directly on VISTA enhancers (red). The transfer learning model is shown as a reference (TL, black). For each threshold (x-axis, 0–1), the y-axis shows the percentage of active sequences among predicted positives (predicted score ≥ threshold). **l-** PPV achieved at the prediction cutoffs defined in panel g, for the three approaches described in panel **k** (see color legend). The absence of red bars for limb and midbrain indicates that, when using randomly initialized models, no sequence reached the stringent thresholds defined in **g**.

We next fine-tuned these sequence-to-accessibility models using experimentally validated embryonic mouse and human enhancers from the VISTA Enhancer Browser^13^ through the process of transfer learning^5,14,15^. As a design-oriented study, we prioritized precision to minimize false positives among the small number of synthetic sequences that can be tested using low-throughput, resource-intensive transgenic reporter assays in vivo. We therefore evaluated model performance using the positive predictive value (PPV), which should reflect the expected success rate among the designed enhancers. Despite the limited number of validated tissue-specific enhancers (between 311 and 432 per tissue; Extended Data Fig. 1d), the resulting sequence-to-activity models achieved high PPVs, reaching 70.6% and above for enhancer sequences not used during training (neither pre-training nor transfer learning; see Fig. 1d and Extended Data Fig. 2a,b for precision–recall analyses).

Notably, 74.5% of all VISTA enhancers active in the midbrain are also active in at least one other central nervous system (CNS) subregion such as forebrain, hindbrain or neural tube, and 22.7% are active in the entire CNS (pan-CNS; Extended Data Fig. 2c,d). Consequently, enhancers of all other CNS subregions scored highly by the midbrain model, with pan-CNS enhancers reaching maximum scores (Extended Data Fig. 2e,f). These results suggest that the midbrain model can be used to design pan-CNS enhancers.

In addition to reaching high PPVs on the VISTA-derived test set, the fine-tuned heart, limb and CNS models successfully reject two additional sets of control sequences, 300,000 randomly generated sequences as well as 300,000 randomly sampled inaccessible genomic sequences (Extended Data Fig. 2g), even though a small subset of random sequences containing tissue-specific transcription factor (TF) motifs received non-zero scores (Extended Data Fig. 2h–j), consistent with prior work showing that random sequences can have enhancer activity^16^. These results suggest that the models might be able to guide in silico enhancer design with a high likelihood of success. Notably, models trained only on ATAC-seq data or only on validated enhancers (direct training without transfer learning) performed worse, with PPVs dropping between 20.9% and 52.1% depending on the tissue (Extended Data Fig. 2k,l), underscoring the value of a two-step transfer learning approach.

**Fig. 2:**
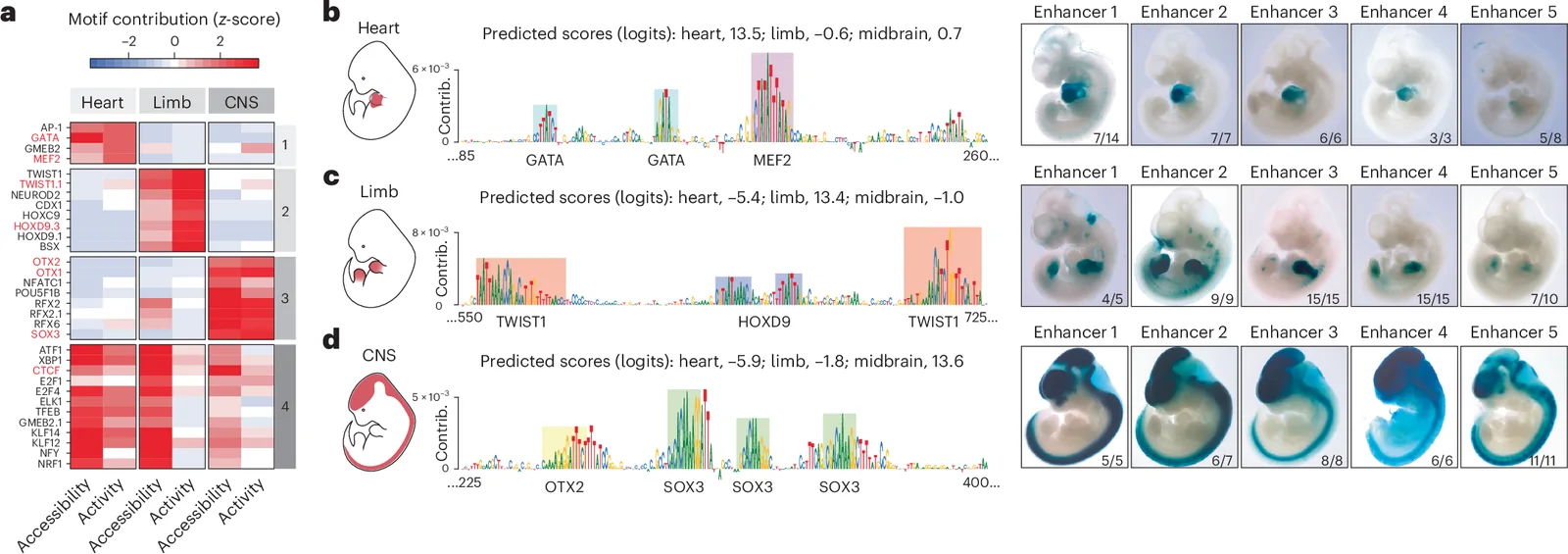
In vivo validation of tissue-specific synthetic enhancers in mouse embryos. **a**, Scaled motif contribution scores before (sequence-to-accessibility) and after (sequence-to-activity) transfer learning (*x* axis; shades of red and blue; see color bar). Motifs discussed in the text are highlighted in red. **b**–**d**, Left: schematic view of E11.5 mouse embryos with heart (**b**), limb (**c**) and CNS (**d**) highlighted in red. Center: nucleotide contribution (contrib.) scores at representative regions (*x* axis) of designed synthetic enhancers (total length, 1,001 bp), with key TF motifs highlighted as colored boxes. Right: representative LacZ-stained transgenic E11.5 mouse embryos carrying five distinct synthetic enhancer sequences per tissue. The leftmost image (enhancer 1) corresponds to the sequences illustrated in the center panel. For each enhancer, the number of embryos showing detectable activity in the target tissue and the total number of transgenic embryos are shown. Following VISTA convention^15^, synthetic enhancers that show consistent reporter gene expression among at least three embryos were considered positive; all embryos are shown in Extended Data Fig. 5.

**Extended Data Fig. 3:**
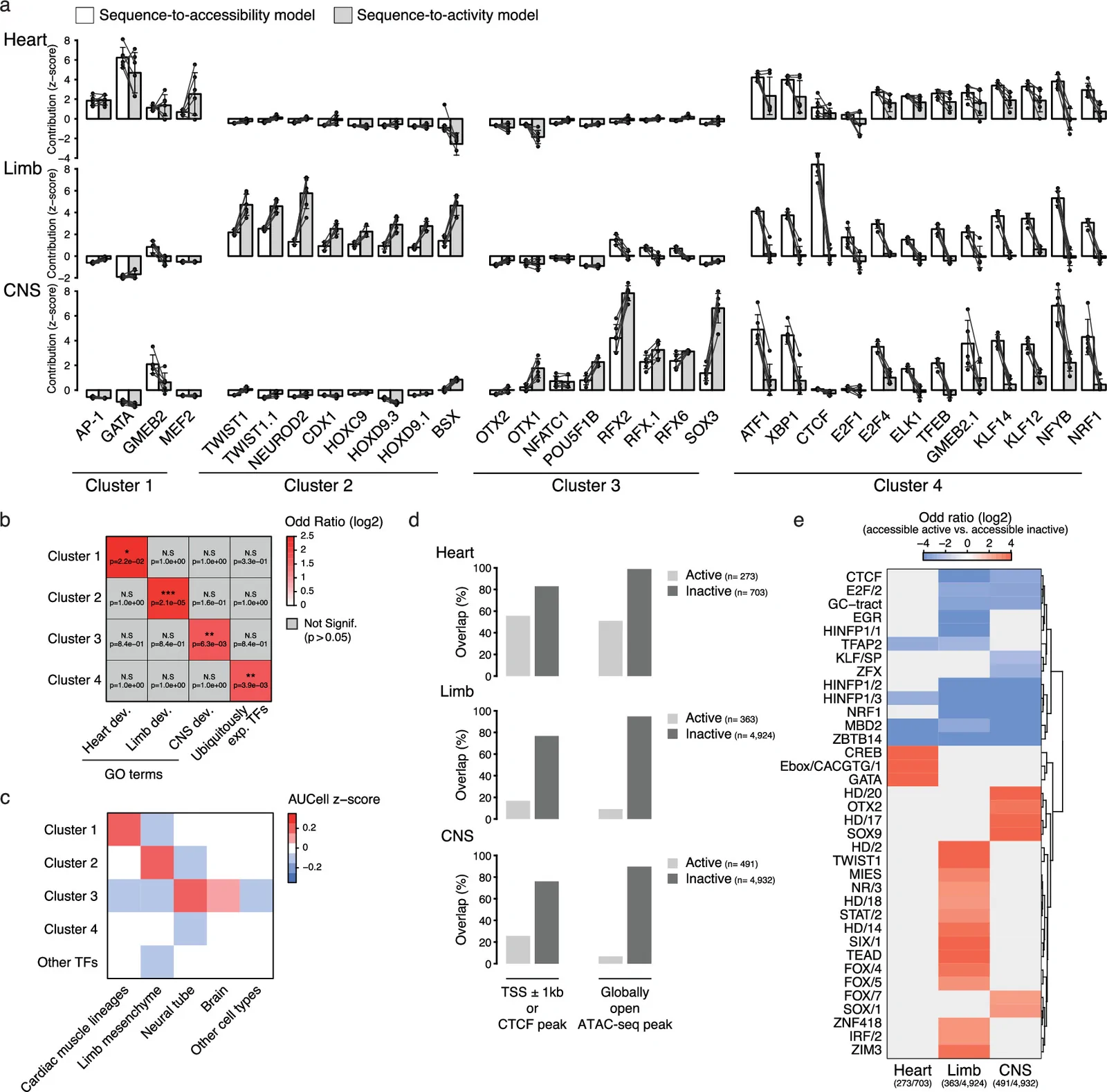
Motif contributions and tissue specificity of TFs and predicted regulatory sequences. **a-** Scaled contribution scores of the important motifs shown in Fig. 2a, for each of the three folds and two replicates (n = 6 total, black dots) of sequence-to-accessibility (white) and sequence-to-activity (grey) models per tissue (see labels on the left). Bar heights indicate mean values and whiskers the standard deviation. **b-** For each motif cluster defined in Fig. 2a, corresponding TF genes were retrieved, and their over-representation across GO categories (heart development, limb development, CNS development) and ubiquitously expressed TF genes (housekeeping) was assessed using one-sided Fisher’s exact test, with multiple comparisons adjusted using the Benjamini–Hochberg FDR method. The color legend indicates the log2(odd ratios), while P values adjusted for multiple testing are shown in plain text. **c-** Single-cell RNA-seq-derived AUCell z-scores for each TF motif cluster defined in Fig. 2a (see Methods). **d-** Percentage of overlap between sequences that were predicted to be accessible and active (light grey) or accessible but inactive (dark grey) and annotated promoters (TSS ± 1 kb) or CTCF peaks (left bar) or globally open ATAC-seq peaks (that is peaks that are accessible in all three tissues; right). **e-** Hierarchical clustering of motifs (Euclidean distances) that were significantly over-represented in sequences that were predicted to be accessible and active versus accessible but inactive.

Furthermore, transfer learning changed the contribution scores of various motifs, reflecting shifts in importance between sequence-to-accessibility versus sequence-to-activity models, and these changes were reproducible between folds and replicates (Extended Data Fig. 3a). A subset of motifs shows similarly high—or even increased—contribution scores after transfer learning, while others show decreased scores, and these changes group motifs into four clusters (Fig. 2a; for the complete list of motifs and associated scores, see Supplementary Table 1). Specifically, motifs with maintained or increased importance (clusters 1–3) correspond to TFs enriched for developmental Gene Ontology (GO) terms of the respective tissue and preferentially expressed in that tissue^17^ (Extended Data Fig. 3b,c), including the master regulators MEF2 for heart^18^, TWIST1 for limb^19^ and SOX3 for CNS^20^. By contrast, motifs in a fourth cluster decrease in importance across all tissues after transfer learning, and their cognate TFs are significantly enriched for broadly expressed TFs without preference for any of the three tissues (Extended Data Fig. 3b,c), such as CTCF, which primarily functions at insulators^21^. Consistently, the sequence-to-accessibility and sequence-to-activity models scored genomic sequences differently, and sequences predicted to be accessible and active versus sequences predicted to be accessible but inactive showed characteristically different genomic locations, accessibility patterns according to ATAC-seq, and TF motif content (Extended Data Fig. 3d,e). These trends further support the role of transfer learning in fine-tuning sequence-to-accessibility models towards predicting enhancer activity.

**Extended Data Fig. 4:**
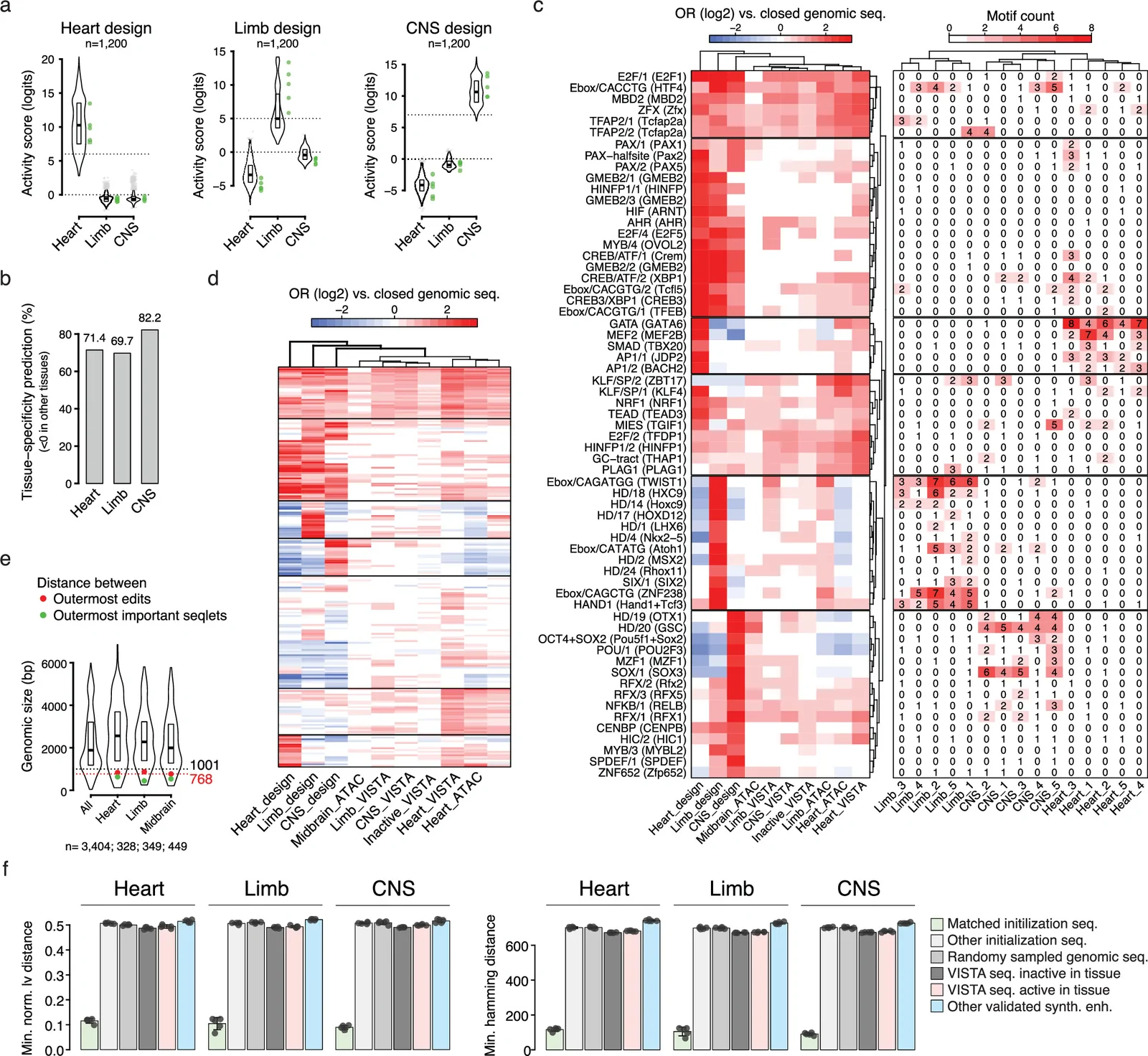
Characterization of designed enhancer sequences. **a-** Predicted activity scores (logits) for synthetic enhancer sequences designed for heart (left), limb (center), or CNS (right), using the three sequence-to-activity models (x-axes). The dotted lines indicate the cutoffs used to define sequences predicted to be strongly active in the target tissue (>6, >5, and >7 for heart, limb, and midbrain, respectively) and inactive in the two other tissues (<0). Sequences chosen for validation are shown as green dots. Box plots show the median (center line), interquartile range (box), and whiskers extending to the most extreme data points within 1.5× the interquartile range (Tukey box plots) with outliers shown as grey dots. **b-** Percentage of sequences that are predicted to be strongly active in their target tissue (see previous panel) and inactive (<0) in the two other tissues. **c-** Left: hierarchical clustering of the top significantly over-represented motifs in designed enhancer sequences, inactive or active VISTA sequences, and ATAC-seq peaks accessible in each of the three target tissues (see x axis labels). Right: corresponding motif counts within the 15 validated synthetic enhancers (see x axis labels). **d-** Hierarchical clustering (Euclidean distances, complete linkage, see dendrogram on top) of designed synthetic enhancers, VISTA enhancers, ATAC-seq peaks, and VISTA inactive control sequences based on their motif enrichments. **e-** Length distribution of VISTA sequences active in heart, limb or midbrain (x axis labels) versus the lengths of validated enhancers (black) and the distance between outermost Ledidi edits (red) or important seqlets (z-score>1, in green). Tukey box plots without outliers. **f-** Normalized Levenshtein distances (left) and Hamming distances (right) between validated synthetic enhancers and other sets of sequences (see color legend). In order, these include the matched initialization sequence from which the enhancers were derived (1 per enhancer), other random initialization sequences (n = 1,200), randomly sampled genomic sequences (n = 1,000), VISTA sequences that are inactive (n = 2,895, 2,873, 2,774) or active (n = 311, 333, 432) in heart, limb and midbrain, respectively; and the other validated synthetic enhancers (n = 15). Bars show the mean, whiskers ±1 sd.

Altogether, these results prompted us to design synthetic enhancers de novo for all three tissues using Ledidi, a gradient-based, model-guided sequence design framework^22^. We used both sequence-to-accessibility and sequence-to-activity models to jointly guide the design of sequences with both high predicted accessibility and activity for each tissue. For each of the three tissues, the majority of the designed enhancers with high scores in their target tissue had low scores in the two other tissues (≥69.7%) and were deemed probably tissue-specific (Extended Data Fig. 4a,b).

**Extended Data Fig. 5:**
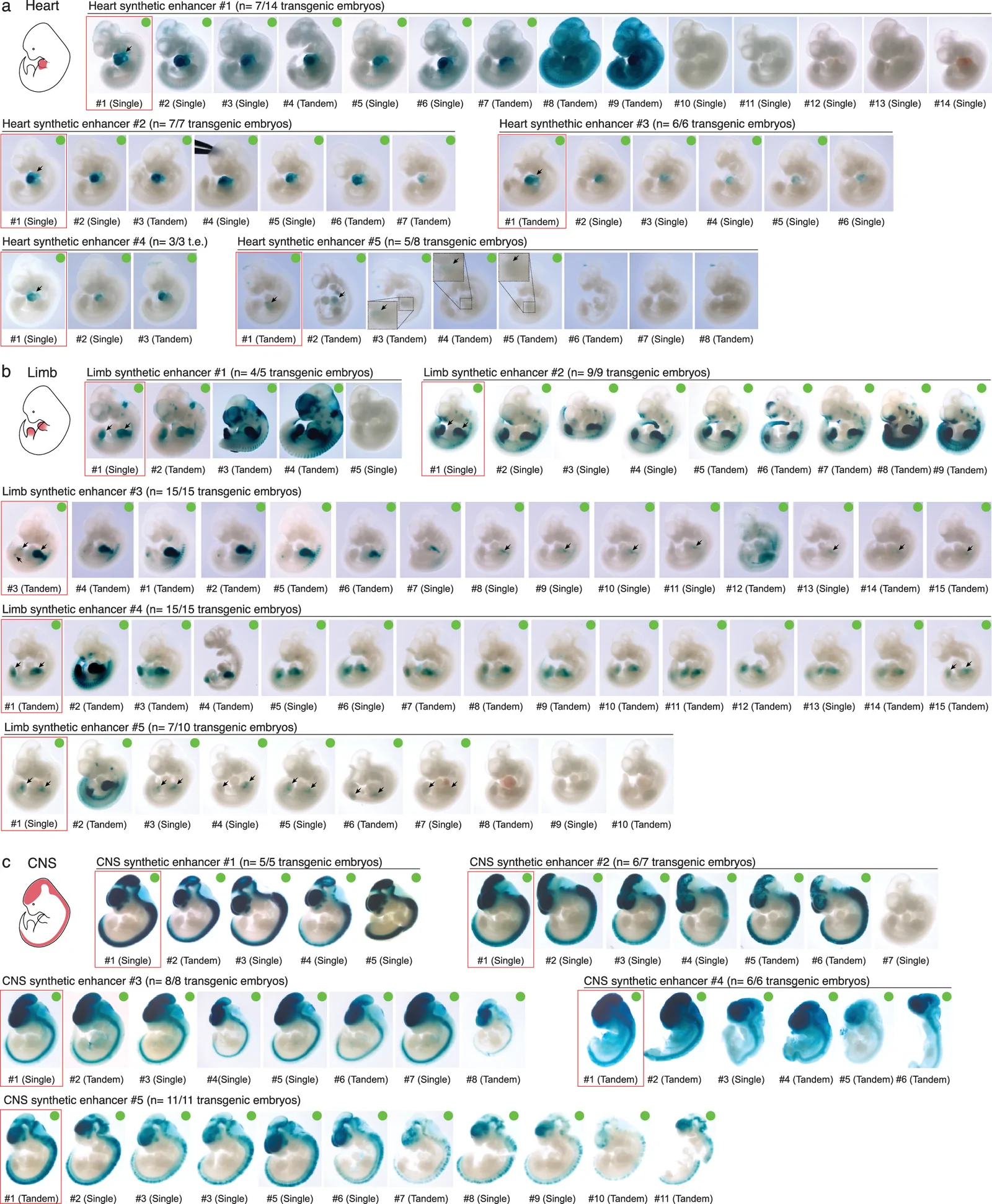
Enhancer reporter assay results for all correctly genotyped E11.5 mouse embryos. **a-** On the top left, a schematic view of E11.5 mouse embryos highlighting the position of the heart (in red) is shown. For each of the five heart synthetic enhancers that were tested in vivo, all corresponding lacZ-stained E11.5 mouse embryos are shown. Following VISTA convention (ref. ^13^), a sequence was considered reproducibly active in the target tissue if at least three embryos carrying either a single copy or a tandem repeat of the reporter constructs (see bottom labels) showed detectable staining in that tissue. Positive embryos are highlighted using a green circle on the top right part of the image. Red boxes indicate the representative embryos that are shown in Fig. 2b–d and are reproduced here for completeness. **b-c-** Same as panel a but for limb and CNS tissues (n = 5 synthetic enhancers per tissue).

We selected five diverse candidate sequences designed to be active in the limb, heart and CNS (15 total; Supplementary Table 2), and accordingly contained key tissue-specific motifs (Extended Data Fig. 4c). Importantly, these synthetic enhancers did not share any significant sequence similarity to the mouse or human genomes (BLAST E-value > 0.05) and are also distinct from VISTA enhancers according to several measures of sequence similarity (Extended Data Fig. 4d–f). We then assessed their enhancer activities in E11.5 mouse embryos using site-specific transgenic reporters without background activity^23^. All 15 synthetic enhancers are reproducibly active in the respective target tissue, with at least three independent transgenic embryos showing detectable signal in the target tissue (Fig. 2b–d and Extended Data Fig. 5). Moreover, four out of five synthetic heart enhancers are exclusively active in the heart, and one (enhancer 5) shows weaker activity in the heart and some off-target activity in the brain (Fig. 2b and Extended Data Fig. 5a). Similarly, four out of five synthetic CNS enhancers are CNS-specific, while one shows weak activity in the heart (Fig. 2d and Extended Data Fig. 5c). Interestingly, all five limb enhancers are strongly active in the limb and show weaker activity in other mesenchymal tissues expressing TWIST1 (ref. ^19^); that is, craniofacial and trunk mesenchyme (Fig. 2c and Extended Data Fig. 5b).

**Extended Data Fig. 6:**
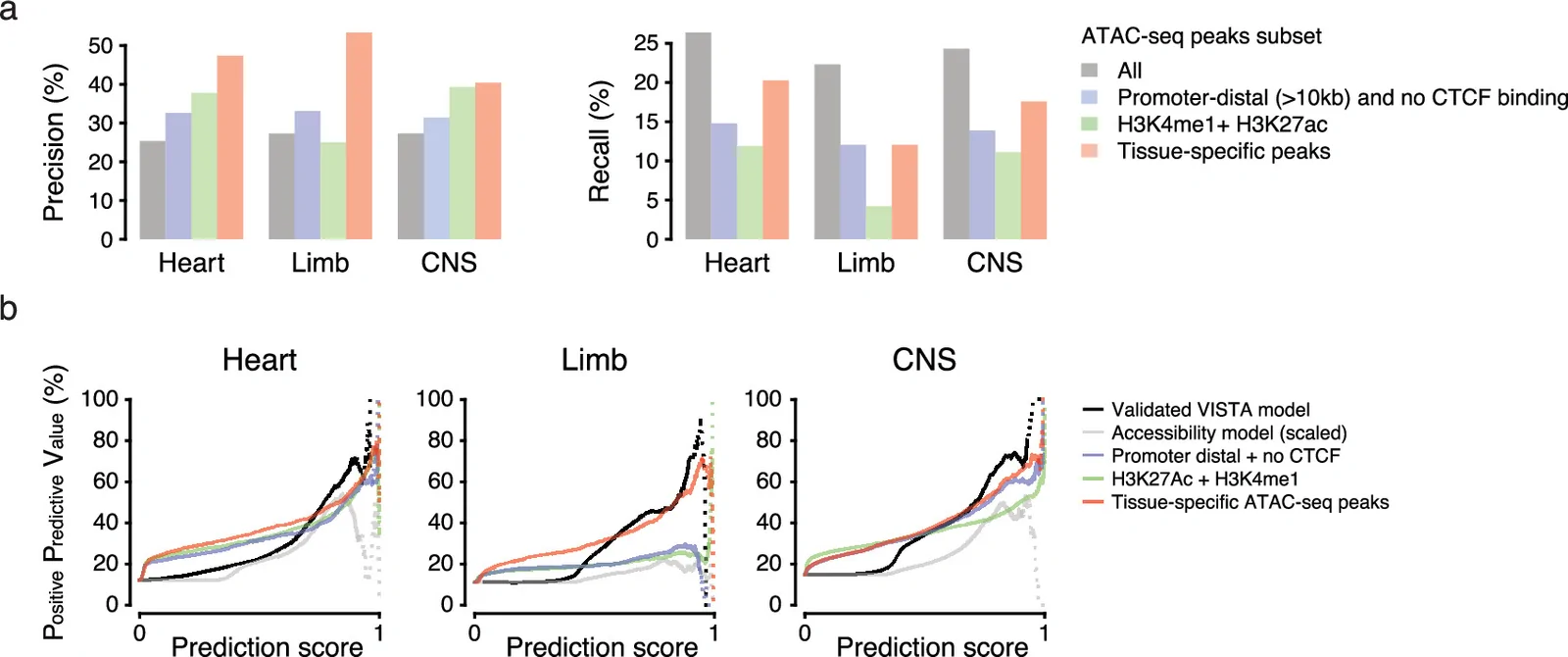
Enhancer prediction using inferred enhancer labels for training. **a-** Predictive performance of established enhancer hallmarks based on genomics data intersection (no modeling), precision (left) and recall (right), benchmarked against VISTA. The tested hallmarks are i) ATAC-seq peak in the target tissue (grey), ii) ATAC-seq peak more than 10 kb away from the closest TSS and devoid of CTCF binding (blue), iii) ATAC-seq, H3K4me1 and H3K27ac peak (intersection) in the target tissue (green), iv) ATAC-seq peak in the target tissue but not in the other two tissues (red, see color legend). **b-** PPV of enhancer activity across prediction score thresholds, comparing the validated sequence-to-activity model fine-tuned on VISTA (black) to models trained using inferred enhancer labels predicted based on the enhancer hallmarks defined in panel a (color legend). Scaled sequence-to-accessibility model is shown as a reference (grey).

For many cell types and tissues, experimentally validated enhancers are rare or absent. To extend our framework for in vivo enhancer design in mammals without relying on such data, we explored whether it can be applied to predicted enhancer activity labels inferred from readily available genomic features. We inferred such labels based on (1) ATAC-seq peaks distal from promoters and CTCF-bound regions; (2) ATAC-seq peaks marked by H3K27ac and H3K4me1 (refs. ^24–26^); and (3) tissue-specific ATAC-seq peaks^27,28^. When assessed for heart, limb and CNS against VISTA, all three strategies enriched for active enhancers compared to accessibility alone (Extended Data Fig. 6a). When used for transfer learning instead of VISTA-derived labels, the inferred labels substantially improved enhancer prediction over the sequence-to-accessibility models and, in some cases, approached the sequence-to-activity models fine-tuned on VISTA (Extended Data Fig. 6b). Among these approaches, tissue-specific ATAC-seq peaks performed particularly well, and such data are broadly available across different tissue contexts using bulk or single-nucleus ATAC-seq approaches^12,28^. Together with prior successful use of differential single-nucleus ATAC-seq signals for in vivo enhancer design in *Drosophila* and zebrafish^6,10^, our cross-validation results for mouse heart, limb and CNS suggest that inferred-label models could design synthetic mammalian enhancers with success rates of at least 60% (Extended Data Fig. 6b).

**Fig. 3:**
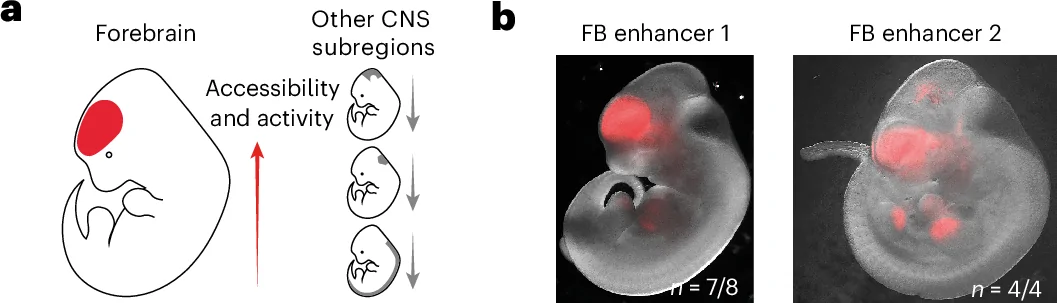
Design and validation of forebrain-specific synthetic enhancers. **a**, Schematic overview of the contrastive approach used to design forebrain-specific synthetic enhancers. Sequences were designed to have high accessibility and activity in the forebrain, and low accessibility and activity scores in the three other CNS subregions (midbrain, hindbrain and neural tube). **b**, Transgenic E11.5 mouse embryos (overlay of brightfield and red channel) carrying a dual reporter construct in which a synthetic forebrain enhancer was placed upstream of mCherry (red). Two different forebrain enhancers were tested; *n* indicates the number of positive embryos among all recovered embryos that carry the construct (see Extended Data Fig. 8d for all recovered embryos and the results for synthetic midbrain enhancer testing).

As a proof of principle for designing subregion-specific enhancers in mammalian tissues, we chose the CNS. We designed two synthetic enhancers for the forebrain and two for the midbrain by requiring high accessibility and activity scores in the target subregion and low scores in the other CNS subregions^22^ (Fig. 3a, Extended Data Fig. 8a,b and Supplementary Table 4). Consistent with prior results in the *Drosophila* embryonic CNS^5^ and the extensive overlap of enhancer activities between CNS subregions in mouse embryos (Extended Data Fig. 2c), achieving subregion specificity proved more challenging than designing enhancers at the whole-tissue level: although both forebrain enhancers were active in the forebrain, one showed substantial off-target activity predominantly in the limb (which was not counter-selected during design). Of the two midbrain enhancers, one was entirely inactive, and although the second showed sporadic activity in the midbrain in two out of eight embryos (Extended Data Fig. 8c,d), the result was not interpretable owing to a 419 bp duplication in the reporter construct (Extended Data Fig. 8e). Therefore, by designing sequences with high forebrain-model scores while counter-selecting against the other CNS subregions, enhancer activity could be substantially decreased in other CNS subregions, particularly the spinal cord, and largely restricted to the forebrain. Together, these results demonstrate that subregion-specific enhancer design in mammals is feasible in principle and will further benefit from dedicated datasets and models that capture regulatory distinctions within the CNS and other complex tissues, ultimately enabling the targeting of individual subregions and cell types.

**Extended Data Fig. 7:**
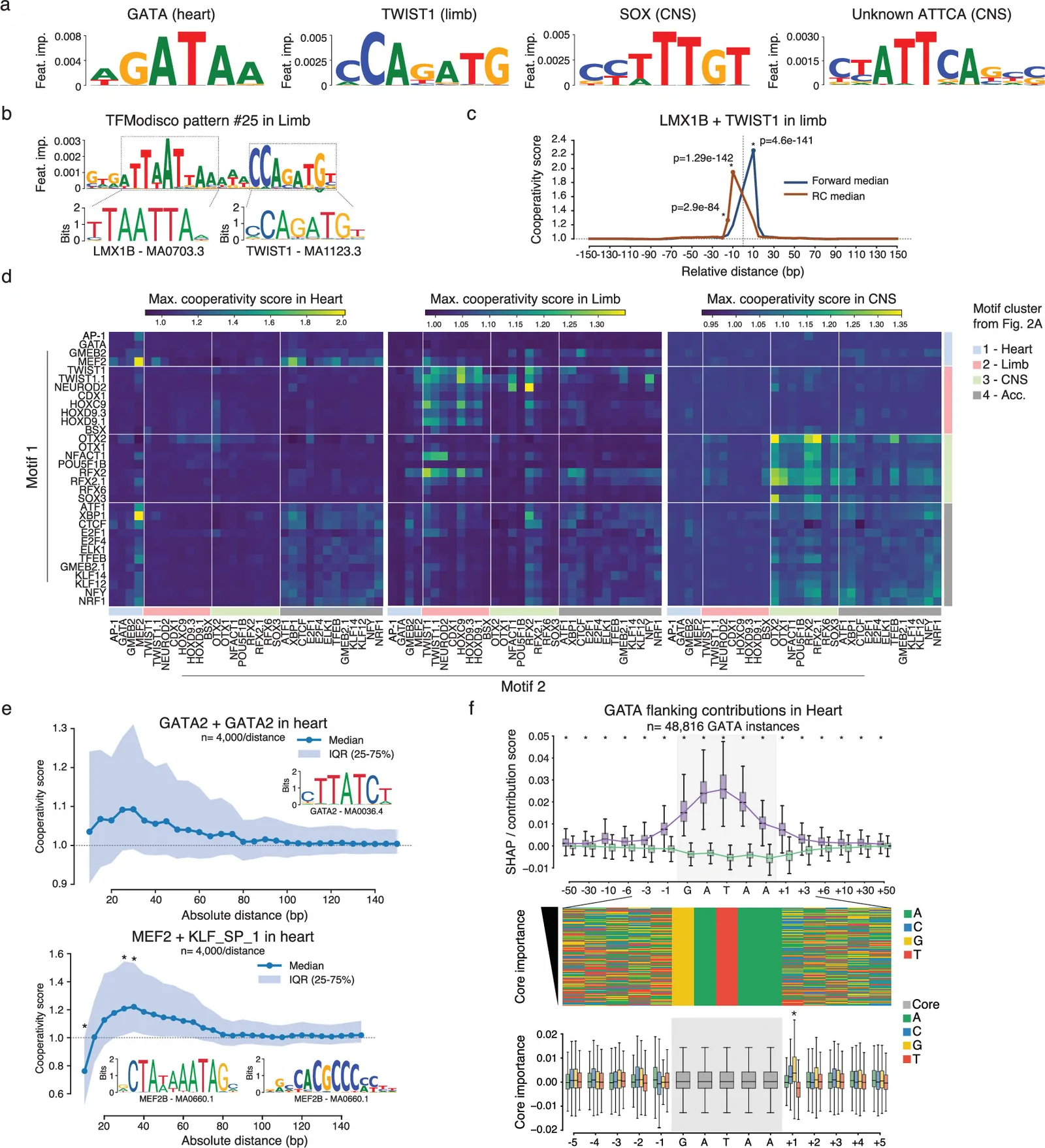
Motifs and higher-order motif grammar learned by models. **a-** Examples of high-contribution patterns identified by TF-Modisco in heart, limb and CNS sequence-to-accessibility models, with the associated TF when a significant hit was found in the JASPAR motif database. **b-** Example of a composite LMX1B/TWIST1 motif identified by TF-Modisco. **c-** Cooperativity between LMX1B and TWIST1 motifs as a function of the orientation of TWIST1 and its distance from LMX1B (placed at center in forward orientation). Two-sided Mann–Whitney U test: * = FDR-adjusted p < 0.05 & |Forward median - RC median |>= 0.2. **d**- Cooperativity between motif pairs. For each pair of motifs from Fig. 2a, the maximum cooperativity score reached at optimal inter-motif distance is shown. **e-** Motif cooperativity at increasing inter-motif distances for a GATA-GATA and a MEF2-KLF motif pair (heart model). Dots show the median values (dark blue) and error bands the IQR (25–75%, light blue). **f-** Impact of flanking nucleotides on GATA motif importance in heart sequence-to-activity model. Shown are attribution scores of the most highly versus the most poorly scoring 10% of GATA-motif instances, irrespective of nucleotide identity (top) and the nucleotide identity next to highly versus poorly scoring GATA-motif instances (bottom). For all patterns identified by TF-Modisco and analyses of higher-order motif syntax, see Supplementary Table 3. Box plots show the median (center line), interquartile range (box), and whiskers extending to the most extreme data points within 1.5× the interquartile range; outliers are not shown.

Our findings establish that mammalian enhancer function is sufficiently encoded in DNA sequence to be learnable and enable de novo design of tissue-specific enhancers that function in vivo. As the models distinguish functional enhancers from other accessible regions, inaccessible regions and random synthetic sequences (Fig. 1d and Extended Data Fig. 2g), they probably captured bona fide sequence-level regulatory features rather than simply memorizing chromatin state. Moreover, the models recover de novo relevant TF motifs and aspects of higher-order TF motif grammar (Extended Data Fig. 7 and Supplementary Table 3). These include known motifs (for example GATA, TWIST1 and SOX) and patterns that do not match known TF motifs, such as an ATTCA-like motif in CNS (Extended Data Fig. 7a; see Supplementary Table 3 for all discovered patterns) and an apparent LMX1B–TWIST1-composite coordinator motif^19^ in limb (Extended Data Fig. 7b,c). In addition, the models learned model-specific cooperativity between TF motif pairs (Extended Data Fig. 7d) as well as preferences regarding motif–motif distances and motif-flanking nucleotides, which are consistent with grammar rules we previously identified and experimentally validated in an unrelated system^4^ (for example, GATA motif spacing and flanking sequences; Extended Data Fig. 7e,f). We provide these analyses in full detail in Supplementary Table 3.

Our study also shows that enhancer design in mammals does not require large, resource-intensive models or massive MPRA-scale datasets. Instead, compact convolutional neural networks (CNNs) combined with genome-wide chromatin profiles that can be readily generated from any sample and with only a few hundred bona fide enhancers per tissue are sufficient for robust design. Moreover, genomic enhancer features can substitute validated enhancers for transfer learning, at least to some extent. As in vivo enhancer assays continue to scale^29,30^, our approach will be widely applicable to the design of more versatile and precise synthetic enhancers across mammalian tissues, cell types and dynamic cell states.

This study focuses on the design of individual enhancer elements for a standardized reporter and promoter context. Enhancer output in native loci can depend on promoter identity, chromatin environment and higher-order genomic context, including auxiliary elements^31,32^. In addition, by focusing locally on individual elements, our approach does not address extended multi-enhancer gene regulatory architectures globally—which long-sequence genome models^33–37^ aim for—or cell types and developmental dynamics in addition to the tissues and stage examined here. Finally, we explored a limited solution space at high specificity and low recall; higher-throughput enhancer-testing approaches that can tolerate a high(er) false-positive rate could, in the future, explore a larger space of possible solutions. These limitations define the scope of this work and—together with other aspects not covered here, including defined enhancer strengths, arbitrary enhancer sequence lengths or complex multi-tissue activity patterns—point to possible future extensions of the framework.

## Methods

### Ethics statement

This study was conducted in accordance with all relevant ethical regulations. All animal procedures were conducted in accordance with National Institutes of Health (NIH) guidelines and were approved by the Institutional Animal Care and Use Committee at the University of California, Irvine, under protocols AUP-23-005 and AUP-26-001.

### Processing of ATAC-seq chromatin accessibility data

#### Peak calling

ATAC-seq mapped reads (mm10) from day 11.5 mouse embryos were obtained as BAM files from previous work^12^ (Supplementary Table 5), and biological replicates were merged using samtools^38^ merge (v.1.9). Peaks were called per biological replicate and on merged reads using MACS2 (ref. ^39^) (v.2.2.5) with the following parameters: -g mm -f BAM -nomodel -shift −100 -extsize 200 -keep-dup all, and bedgraph pileup files were converted to bigwigs using the bedGraphToBigWig function^40^. Only confident peaks called in the two biological replicates and with merged reads were retained.

#### Peak annotation

Confident ATAC-seq peaks from heart, limb and CNS subregions (midbrain, forebrain, hindbrain, neural tube) were merged and resized to 1,001 bp (mean summit ±500 bp). Mean signal was computed from the corresponding merged bigwig files and log_2_ scaled as: log_2_(signal / sum(signal across all regions in that tissue) × 1 × 10^6^). Outlier regions (scaled signal of >99.95th percentile) were removed, and the remaining regions were clustered using a two-layer self-organizing map (kohonen R package (v.3.0.10)^41^, supersom) on a 4 × 5 hexagonal, toroidal grid (layer 1, peak presence/absence per tissue (binary); layer 2, log_2_-scaled ATAC-seq signal). Peak clusters were further classified into ten meta-clusters, including a cluster of ‘globally open’ regions (accessible in all tissues, *n* = 17,212) as well as heart-specific, limb-specific and midbrain-specific clusters (*n* = 3,008; 1,407 and 2,551, respectively). Weak clusters with no clear pattern were removed (*n* = 5,482). The full list of annotated peaks (*n* = 40,196) is available in Supplementary Table 6.

### Deep learning data preparation

#### VISTA sequences

In vivo enhancer activity data were downloaded from the VISTA Enhancer Browser^13^ on 1st September 2024. Sequences corresponding to mutated variants or with length >5 kb were removed, while sequences shorter than 1,501 bp were resized to a minimum length of 1,501 bp (*n* = 3,206; Supplementary Table 7).

#### Inaccessible control regions

The mm10 version of the mouse genome was binned into contiguous 1,001 bp bins, and bins located <1.5 kb away from the closest ATAC-seq peak or VISTA sequence were removed (for VISTA sequences of human origin, the mm10 coordinates of their orthologous regions were used; Supplementary Table 7). Then, one million bins were randomly sampled to serve as a pool of negative control regions.

#### Cross-validation scheme

The genomic coordinates of ATAC-seq peaks (*n* = 40,196), VISTA sequences (*n* = 3,206) and inaccessible control regions (*n* = 1,000,000) were combined and split into 20 strictly non-overlapping folds, ensuring a minimum gap of 1.5 kb between sequences assigned to different folds (for VISTA sequences of human origin, the mm10 coordinates of their orthologous regions were used; Supplementary Table 7). We then used a cross-validation setup in which 18 folds were used for training, one for validation and one for testing. However, regions on mouse chromosome 18 were systematically excluded from training and validation to serve as a fixed, ‘shared’ test set across all models and tissues.

### Sequence-to-accessibility models

#### Data augmentation

Augmentations were applied after splitting regions into training, validation and test folds (see ‘Cross-validation scheme’). For each ATAC‑seq peak and inaccessible control region, we included five 1,001 bp windows centered from −400 to +400 bp relative to the region center, with a 200 bp stride (−400, −200, 0, +200, +400). For peaks labeled as heart-specific, limb-specific and midbrain-specific (Supplementary Table 6), we specifically reduced the stride to 50 bp in the corresponding tissue (*n* = 17 windows per region). Finally, reverse-complemented sequences were added.

#### Balancing

Inaccessible control regions were downsampled to ~1.5 times the total number of ATAC-seq peaks after augmentation, to increase the proportion of tissue-specific peaks per tissue (~70×), globally open and tissue-specifically closed regions (both ~20×), while reducing the proportion of globally closed regions (~2×) compared to their abundance in the genome. The scheme also balanced region types with different CpG content to mitigate confounding between CpG content and accessibility. The numbers of sequences used for each tissue are available in Supplementary Table 8. See the Data availability section to access the corresponding sequences.

#### Model architecture and training

For each region, DNA accessibility was computed from a merged bigwig file (containing the MACS2 treat pileup read coverage; see above) as log_2_(mean ATAC‑seq signal + 1), and predicted using the DeepSTARR CNN architecture^4^ with minor modifications. The CNN uses one-hot-encoded 1,001 bp DNA sequences (A, (1,0,0,0); C, (0,1,0,0); G, (0,0,1,0); T, (0,0,0,1)) and four 1D convolutional layers (filters, 256,120,60,60; size, 7,3,3,3; padding, same), each followed by batch normalization, a ReLU non-linearity, and max-pooling (size, 3). The convolutional stack is followed by two fully connected layers (64 and 256 neurons), each followed by batch normalization, a ReLU non-linearity activation function, and dropout (fraction, 0.5). The final layer is mapped to the accessibility signal output. The models were implemented and trained in Python (v.3.7.15) using Keras (https://keras.io, v.2.4.3) from TensorFlow^42^ (v.2.4.1) using the Adam optimizer^43^ (learning rate, 0.005) with mean squared error as loss function, a batch size of 128 and early stopping with a patience of five epochs. We did not implement a Tn5 bias model that primarily affects nucleotide-resolution profile predictions but is less relevant for predictions of overall accessibility signal^44^, and we do not observe Tn5-related patterns with high attributions in the models (Supplementary Table 3). To account for variance between different runs, each model was trained in duplicate on three different held-out test folds (total of six models per tissue). For each tissue, models converged and showed negligible variance in predictions, and predictions were averaged across runs.

#### Model performance

Prediction bigwig tracks were generated for chromosome 18, which was excluded from all runs, using a 1,001 bp sliding window with a 200 bp stride. The PCC between measured and predicted ATAC‑seq signal was computed on the union of heart, limb and midbrain ATAC‑seq peaks (Fig. 1b), on the entire held‑out test set for each tissue (Extended Data Fig. 1b), and on delta values between tissue pairs (Extended Data Fig. 1c), emphasizing performance at tissue‑specific peaks. To compare models across tissues, observed DNA accessibility values were *z*-scored across all held-out regions within each tissue, and regions with a *z*-score of >1 in any tissue were clustered using *k*-means (*k* = 6; Fig. 1c). To compute the overlap with mouse gene transcription start sites, mm10 transcription start site coordinates were retrieved from GENCODE vM25 (Extended Data Fig. 1a).

#### Nucleotide contribution scores

We used DeepExplainer (the DeepSHAP implementation of DeepLIFT^45,46^; updated code at https://github.com/AvantiShri/shap/blob/master/shap/explainers/deep/deep_tf.py) to compute nucleotide contribution scores on the subset of held‑out regions exactly centered on ATAC‑seq peak summits (see ‘Cross‑validation scheme’), using only the forward‑strand sequence. For each input, 100 dinucleotide‑shuffled versions were used as reference sequences, and final contribution scores were obtained by multiplying hypothetical importance scores by the one‑hot-encoded matrix of the corresponding sequence. For each nucleotide, scores were averaged across all replicates and folds per tissue (*n* = 6).

### Sequence-to-activity models

#### Data augmentation

Augmentations were applied after splitting regions into training, validation and test folds (see ‘Cross-validation scheme’). VISTA sequences were tiled using a 1,001 bp sliding window with a 50 bp stride. Only windows with ≥950 bp overlap with the original region were retained, and reverse-complemented sequences were added. The final number of sequences used for each tissue is available in Supplementary Table 9. See the Data availability section to access the corresponding sequences.

#### Model architecture and training

To classify DNA sequences based on their in vivo activity, we used a transfer‑learning approach in which weights from the sequence‑to‑accessibility regression models were used to initialize a second CNN in the corresponding tissue^5,14,15^. All layers were kept trainable, and the final layer’s activation was changed to a sigmoid function suitable for binary classification tasks. Models were trained with the Adam optimizer^43^ (with a learning rate of 1 × 10^−4^), binary cross-entropy loss, a batch size of 128 and early stopping with a patience of 20 epochs.

To account for variance between different training runs and improve the accuracy and robustness of the models, each model was trained in duplicate on three different held-out test folds, for a total of six models per tissue. For the three tissues, all models converged and showed negligible variance in predictions, and predictions were therefore averaged across runs.

#### Model performance

For sequence‑to‑activity models after transfer learning, PPV (TP / (TP + FP)) was computed on the held‑out test set across increasing prediction thresholds (that is, sequences were classified as positive when predicted activity was ≥ the threshold; Fig. 1d) in each tissue, and we reported the maximum PPV achieved while ensuring that at least 100 predicted‑positive sequences remained. We also evaluated two alternatives: models trained directly on annotated VISTA enhancers without pre‑training; and predictions from the sequence‑to‑accessibility models (scaled to [0, 1] and used as activity predictions). In these cases, PPV was measured at the prediction thresholds corresponding to the maxima for the transfer‑learning model in each tissue (Extended Data Fig. 1h–j).

#### Nucleotide contribution scores

Nucleotide contribution scores were computed with the approach described for the sequence‑to‑accessibility models, using the forward‑strand sequences of all tiles within the held‑out test set. For each nucleotide, scores were averaged across all replicates and folds per tissue (*n* = 6).

### Design of tissue-specific synthetic enhancers

For each tissue, 1,200 random DNA sequences were generated with dinucleotide frequencies matched to VISTA sequences and used as input for Ledidi^22^ (v.2.1.0), a model‑guided gradient optimization approach. To limit the number and magnitude of edits, the edit‑penalty parameter was set to 0.1. Seed sequences were then optimized using both the sequence‑to‑accessibility and sequence‑to‑activity models, with target predicted values set to 12 and 14, respectively. This creates individual sequences with both high predicted accessibility and high predicted activity as candidate enhancers. For activity optimization, the final sigmoid layer was removed, and pre‑sigmoid logits were used to avoid gradient saturation and improve optimization stability. Designed sequences with a significant match to either the mouse or the human genome were identified using Blastn (through NIH NCBI Blast, https://blast.ncbi.nlm.nih.gov/Blast.cgi) with default parameters (word size, 11; expect threshold, 0.05) and removed, yielding 1,049 usable sequences for heart, 1,020 for limb and 978 for CNS or midbrain. From the designed sequences with high predicted activity in the target tissue (thresholds of >6 for heart; >5 for limb; >7 for CNS and midbrain) and low activity in the other two (<0; *n* = 654, 351 and 787 for heart, limb and CNS or midbrain, respectively; Extended Data Fig. 2c), 15 were selected for in vivo validation (five per tissue). For these 15, we performed BLASTN searches using more sensitive settings (word size, 7 bp and E-value threshold, 0.05) to align them separately against the VISTA sequences and the mouse (mm10) and human (hg19) genomes. None of the 15 sequences showed a significant match in either genome or VISTA. Selected sequences, their predicted activities and the corresponding nucleotide contribution scores are available in Supplementary Table 2.

### Determination of TF motif importance and clustering of important TF motifs

Position weight matrices (PWMs) for transcription factor motifs listed in prior work^47^ were mapped onto the held‑out sequences using the matchMotifs function from the motifmatchr R package^48^ (v.1.26.0; parameters: bg= ‘subject’, p.cutoff= 5 × 10^−5^), and mean nucleotide contribution scores were computed for each motif instance with respect to the sequence‑to‑accessibility or sequence‑to‑activity models. These per‑instance means were then averaged per PWM (*n* = 2,174) and *z*‑scored per tissue (see sheet 2 of Supplementary Table 1), and only PWMs with a *z*‑score of ≥2 for either accessibility or activity in any of the three tissues were retained (*n* = 358). Redundant PWMs were then filtered using the non‑redundant motif clusters described in a previous publication^47^, selecting only the PWM with the highest *z*‑score as the representative of each cluster. Accessibility and activity *z*‑scores were clipped at the 5th and 95th percentiles for each tissue, clustered with the hclust function in R (v.4.4.1) using Euclidean distances, and the resulting tree was cut into four clusters with the cutree R function (Fig. 2a).

### GO enrichment analysis

Mouse gene IDs associated with heart (GO:0007507), limb (GO:0060173) and CNS (GO:0007417) development were retrieved from the org.Mm.eg.db R package (v.3.19.1). The list of ubiquitously expressed housekeeping genes was retrieved from a previous publication^49^. For each of these four categories, the overrepresentation of the TFs associated with the four clusters defined in the previous section (*n* = 12, 55, 28, 56; Fig. 2a) was assessed using Fisher’s exact test, using all TFs represented in the PWM database as background (*n* = 599). *P* values were corrected for multiple testing using the false discovery rate (FDR) method (significance threshold: FDR < 0.05).

### Single-cell RNA-seq analyses

Mouse single‑cell RNA‑seq data were retrieved from GSE119945, and only cells from day 11.5 embryos were considered (*n* = 602,722). We then selected the TF genes associated with the PWMs database described earlier (*n* = 573 after intersecting with the expression matrix), and cells not related to any of the target tissues (that is, cardiac muscle lineages, limb mesenchyme, brain or neural tube) were labeled as ‘other cell types’. Per‑cell gene‑level rankings were then computed on the raw count matrix using the AUCell R package (v.1.26.0). Area under the curve scores were finally computed for each of the TF clusters described earlier (Fig. 2a) and for the remaining TFs (regrouped under the ‘other TFs’ label), before being *z*-scored and averaged per cell type for visualization (Extended Data Fig. 3c).

### In vivo validation of tissue-specific synthetic enhancers

To test synthetic enhancer sequence activity in mouse embryos, each DNA sequence was custom ordered as a gBlock (IDT) (see Supplementary Table 2 for sequences). Synthetic enhancers were then inserted into a PCR4-Shh::lacZ-H11 vector backbone (Addgene, 139098) using Gibson assembly. Sequence identity was confirmed by enzymatic digestion and Sanger sequencing. A mix of Cas9 protein (final concentration of 20 ng μl^−1^; IDT, 1074181), sgRNA (50 ng μl^−1^) and donor plasmid (7 ng μl^−1^) was prepared in injection buffer (10 mM Tris, pH 7.5; 0.1 mM EDTA) and injected into the pronucleus of FVB embryos. Mice (FVB and CD-1 strains) were kept in standard housing conditions (temperature of 19–23 °C and humidity of 40–60%) with food and water provided ad libitum on a reversed 12 h dark–light cycle. Pregnant dams were humanely killed, and E11.5 embryos were carefully removed under brightfield stereoscopes in ice-cold PBS (Cytiva, SH30256.01). LacZ staining was carried out as previously described, using the same standard protocol as for all VISTA enhancers^13,50^. Embryos were collected at E11.5 and fixed in 4% paraformaldehyde for 30 min, followed by three washes (30 min each) in embryo wash buffer (2 mM MgCl_2_; 0.01% deoxycholate; 0.02% NP-40; 100 mM phosphate buffer, pH 7.3). Embryos were then incubated overnight in X-gal staining solution (0.8 mg ml^−1^ X-gal; 4 mM potassium ferrocyanide; 4 mM potassium ferricyanide; 20 mM Tris, pH 7.5, in wash buffer) to visualize LacZ activity. The following morning, embryos were rinsed three times for 10 min with PBS and then fixed again in 4% paraformaldehyde. Images were captured using a ZEISS Stemi 508 microscope with an Axiocam 208 digital camera. Both sexes of embryos were presumed to be included. Yolk sacs were collected for genotyping, and successful integration events at the H11 locus were determined by PCR using primers described previously^23^. All embryos with single-copy and tandem (multiple-copy) transgene integrations at the H11 locus were considered in the analysis (see Extended Data Fig. 5 and previous work^23^ for details). Scoring of tissue positivity was performed for each embryo by two independent scorers in a non-blinded fashion.

### In vivo validation of midbrain and forebrain-specific synthetic enhancers

**Extended Data Fig. 8:**
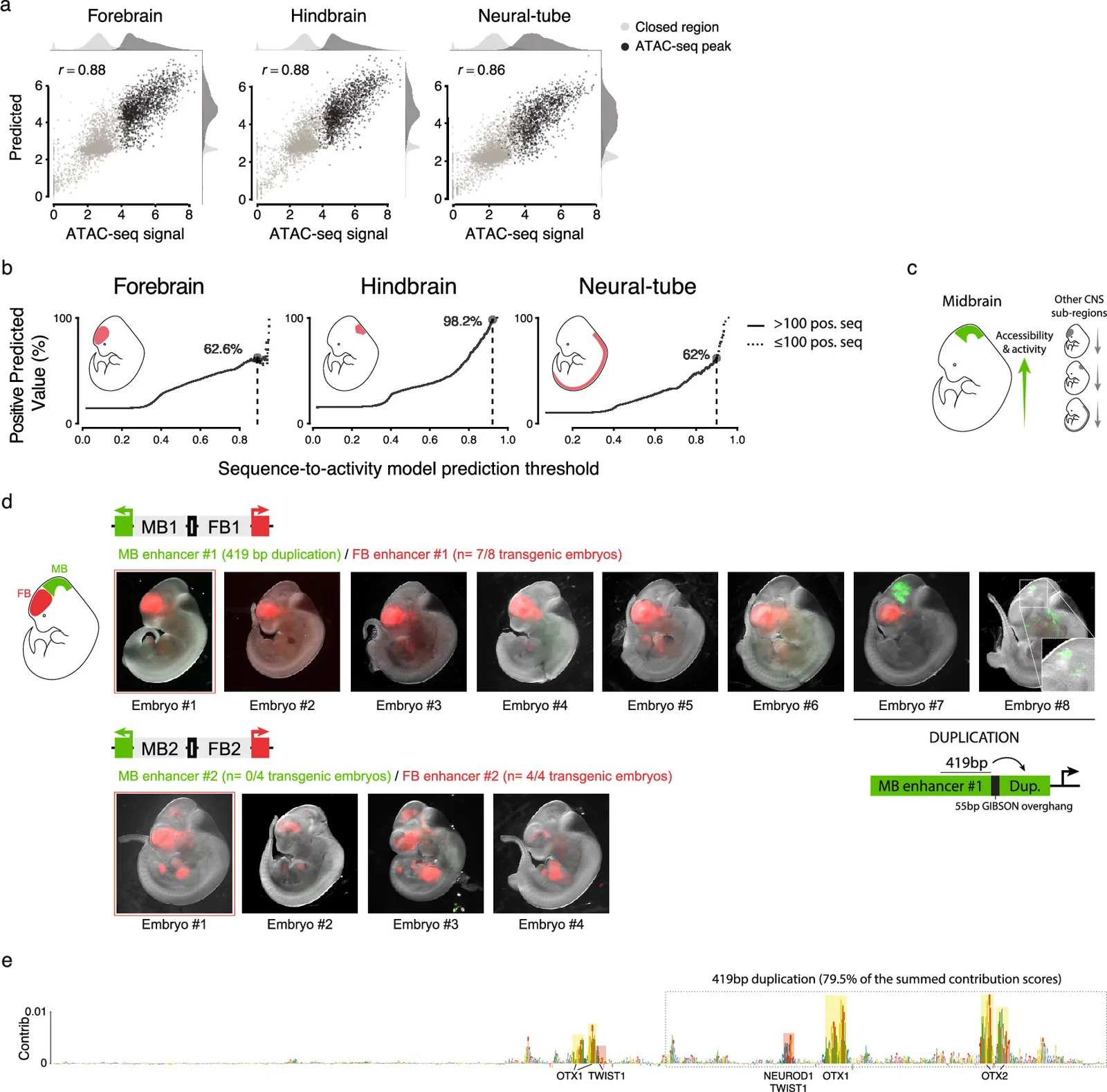
Proof-of-principle for designing CNS subregion-specific enhancers. **a-** Observed vs. predicted ATAC-Seq signal on sequences that were not used for training (test set) for forebrain, hindbrain and neural-tube sequence-to-accessibility models. Number of sequences n = 260,359; 259,171; 243,739 for forebrain, hindbrain and neural-tube, respectively. **b-** PPV of enhancer activity predictions across prediction score thresholds for forebrain, hindbrain and neural-tube sequence-to-activity models. For each threshold (x-axis, 0–1), the y-axis shows the percentage of active enhancers (positives) among all predicted enhancers (predicted score ≥ threshold). The maximum value is reported when at least 100 positive sequences remain (dashed line). **c-** Schematic overview of the contrastive approach used to design midbrain-specific synthetic enhancers. Sequences were designed to have high accessibility and activity in the midbrain, and low accessibility and activity scores in the three other CNS sub-regions, which include forebrain, hindbrain and neural tube. Number of sequences n = 31,636. **d-** Schematic view of E11.5 mouse embryos highlighting the position of the forebrain (FB, in red) and the midbrain (MB, in green). Each of the two FB/MB synthetic enhancer pairs were tested in vivo using dual-reporter constructs (top), in which synthetic MB and FB enhancers were placed upstream of GFP (green) and mCherry (red), respectively, and separated by an insulator sequence (black). All E11.5 mouse embryos containing a single copy of the reporter construct are shown in overlays of brightfield, red and green channels. Red boxes indicate the representative embryos that are shown in Fig. 3b (brightfield and red channel only) and are reproduced here for completeness. **e-** Nucleotide contribution scores of the MB1 synthetic enhancer, with key TF motifs highlighted as colored boxes. The dashed box (right) indicates the 419 bp duplication found in the reporter construct, rendering the corresponding results for MB1 in panel d non-interpretable.

FB1–mCherry/MB1–eGFP and FB2–mCherry/MB2–eGFP dual constructs were made by inserting these synthetic enhancer sequences (Supplementary Table 4), ordered as gBlocks, into the dual-enSERT-2.2 plasmid^51^ (Addgene, 211942) using Gibson Assembly cloning (New England Biolabs). Sequence identity was confirmed by enzymatic digestion and whole-plasmid sequencing (Plasmidsaurus). For MB1, this revealed a 419 bp duplication, rendering the results for MB1 non-interpretable (see main text and Extended Data Fig. 8). Transgenic mice carrying enhancer-reporter transgenes were generated using site-directed dual-enSERT transgenesis as previously described^51^. After pronuclear microinjections, F0 embryos were collected at embryonic day E11.5 and prepared for fluorescence microscopy. Both sexes of embryos were presumed to be included. The embryos were genotyped by PCR and Sanger sequencing as previously described^23,51^, and all embryos with a single-copy insertion of the dual-enSERT transgene were included in the analysis. Imaging of dual-enSERT embryos was conducted as previously described^51^. Embryos were imaged using a ZEISS SteREO Discovery.V8 stereoscope equipped with a monochromatic camera (Axiocam 202, Zeiss), fiber optic light source (Zeiss, CL1500) and LED fluorescent laser (X-Cite, Xylis) emitting at 488 (eGFP) and 555 (mCherry) nm wavelengths. Merged images were created using the Zeiss Biolite Software.

### Transfer learning using inferred labels

For each target tissue, ATAC-seq peaks were assigned inferred activity labels using three strategies based on genomic features associated with enhancer activity. First, peaks were labeled as active if they were located more than 10 kb from the nearest annotated transcription start site (GENCODE vM25, mm10) and did not overlap CTCF peaks (defined as the union of E12.5 limb and midbrain peaks obtained from previous work^52^). Second, peaks were labeled as active if they overlapped both H3K27ac and H3K4me1 peaks in the target tissue (obtained from prior work^53^). Third, peaks were labeled as putative active enhancers if they were specifically accessible in the target tissue; that is, they did not overlap ATAC-seq peaks in the other two tissues (Supplementary Table 6). Peaks not meeting the respective criterion were labeled as inactive. When needed, inactive sequences were downsampled so that positive sequences represented at least 13% of the training set, similar to the class balance of the VISTA-based training dataset. These inferred labels were then used for transfer learning as described above for the sequence-to-activity models. To benchmark these labeling strategies, we also applied them to VISTA sequences to assess their baseline performance (Extended Data Fig. 6a).

### De novo motif discovery and analysis of motif syntax rules

De novo motif discovery was performed on feature-attribution profiles from sequence-to-activity models, and motif syntax rules were assessed using in silico motif insertion and genomic motif-instance analyses. Detailed methods are provided in Supplementary Information. Motif annotations, first-layer convolutional kernel matches and motif syntax analysis results are reported in Supplementary Table 3.

### Design of midbrain and forebrain-specific synthetic enhancers

Additional sequence-to-accessibility models were trained for forebrain, hindbrain and neural tube, and corresponding sequence-to-activity models were obtained by transfer learning on VISTA Enhancer Browser sequences, as described above (datasets and genomic regions available in Supplementary Tables 5–9). For each target tissue (forebrain or midbrain), 600 random DNA sequences with dinucleotide frequencies matched to VISTA sequences were generated and used as input for Ledidi^22^ (v.2.1.0). Sequences were optimized jointly across all eight models with an edit-penalty parameter of 0.1, requiring high predicted accessibility and activity in the target tissue while minimizing both predictions in the other CNS subregions. Target values were set to ten for accessibility and 12 for activity in the target tissue, and to two for accessibility and −4 for activity in the non-target CNS tissues. For activity optimization, the final sigmoid layer was removed, and pre-sigmoid logits were used as described above.

Designed sequences with a significant match to either the mouse or human genome were identified using Blastn with the same settings described above and removed, yielding 559 usable sequences for forebrain and 563 for midbrain. From sequences with high predicted activity in the target tissue (>2 for both forebrain and midbrain) and low predicted activity in the other three CNS tissues (<0), two candidates per tissue were selected for in vivo validation. Selected sequences, their predicted activity scores and the corresponding nucleotide contribution scores in the target tissue are available in Supplementary Table 4.

### Statistics and data visualization

All statistical analysis and plots were done in R (v.4.4.1)^54^ using the data.table package^55^.

### Reporting summary

Further information on research design is available in the Nature Portfolio Reporting Summary linked to this article.

## Online content

Any methods, additional references, Nature Portfolio reporting summaries, source data, extended data, supplementary information, acknowledgements, peer review information; details of author contributions and competing interests; and statements of data and code availability are available at https://doi.org/10.1038/s41588-026-02729-1.

## Supplementary information

Supplementary Information Supplementary methods with references.

Reporting Summary

Peer Review file

Supplementary Table 1 Complete list of motifs and scores associated with Fig. 2a.

Supplementary Table 2 Designed synthetic enhancers selected for in vivo validation.

Supplementary Table 3 Higher-order motif syntax rules.

Supplementary Tables 4–9 Supplementary Table 4, Midbrain and forebrain-specific synthetic enhancers selected for in vivo validation; Supplementary Table 5, Publicly available ATAC-seq BAM files used in this study; Supplementary Table 6, Full list of annotated ATAC-Seq peaks; Supplementary Table 7, Full list of VISTA Enhancer Browser sequences; Supplementary Table 8, Number of sequences used for sequence-to-accessibility models; Supplementary Table 9, Number of sequences used for sequence-to-activity models.

## Data Availability

The transcription factor motif database is available at https://www.vierstra.org/resources/motif_clustering. The weights and architecture of the final pre-trained sequence-to-accessibility and sequence-to-activity models are available at https://huggingface.co/Shenzhi-Chen/DeepSTARR-Mouse. The data used to train and evaluate the models are available at https://huggingface.co/datasets/Shenzhi-Chen/DeepSTARR-Mouse-dataset.

## Code availability

All scripts used in this study have been deposited on Zenodo^56^. The Zenodo record includes the code used for model training, predictions, contribution analyses, synthetic enhancer design, data curation and preparation, and downstream analyses. It also contains further information and instructions for locating and using the relevant scripts.
