# Supplementary material for: Predictive design of tissue-specific mammalian enhancers that function in the mouse embryo
Source: Nat Genet. Author manuscript; Available in PMC 2026 Sep 25. (PMC13553301; doi:10.1038/s41588-026-02729-1)
Supplement: Supplementary file 3 [file NIHMS2209045-supplement-Supplementary_Information.pdf]

nature genetics

Supplementary information https://doi.org/10.1038/s41588-026-02729-1

# Predictive design of tissue-specific mammalian enhancers that function in the mouse embryo

In the format provided by the authors and unedited

# Supplementary Methods

## De novo motif discovery analysis

For each sequence-to-activity model, de novo motif discovery was performed using TF-MoDISco (modisco-lite v. 2.4.0) on feature attribution profiles of 14,922 test set sequences, computed with the DeepSHAP implementation of DeepLIFT[45,46]. Motif discovery was run with the ‘modisco motifs’ using max_seqlets = 50000, and discovered patterns were matched to motifs in the JASPAR 2024[57] core vertebrate non-redundant PFMs database supplemented with Tn5 motifs using ‘modisco report’. In addition, for each discovered pattern, the closest-matching kernel in the first convolutional layer of the model was identified. Kernel weights were extracted from the model, and patterns were matched to kernels using the tomtom implementation in memelite. The best-matching kernel for each pattern was reported. q values were corrected for multiple testing using the Benjamini–Hochberg method implemented in false_discovery_control (SciPy v1.14.1). For matching, pattern feature-contribution matrices were normalized to a total sum of 7, corresponding to the kernel length. Kernel weights were converted to per-position nucleotide probability distributions by applying a softmax (torch.nn.Softmax, PyTorch v2.5.1) after scaling with a temperature of 10. Best matches in the JASPAR database and among first-layer convolutional kernels are provided in Supplementary Table 3.

## Analysis of motif syntax rules

For each sequence-to-activity model, motif syntax rule analysis was performed using two sets of motifs: 1) the important motifs described in Fig. 2a (PWMs available in Supplementary Table 1) or 2) the JASPAR PWMs matching the patterns identified using TF-MoDISco ($q < 0.01$, see previous section). For each PWM, positions with a nucleotide probability greater than 0.6 were defined as consensus bases. The consensus sequence was then defined as the region spanning from the 5′-most consensus base to the 3′-most consensus base across the full length of the PWM. Positions within this region that did not contain a consensus base were represented as N in the consensus sequence.

<u>Cooperativity score</u>

To evaluate the cooperativity between motif pairs, we generated a set of random backbone sequences in which pairs of motifs were implanted at specific positions *in silico*, and computed their cooperativity scores as previously described[4]:

$$cooperativity\ score = \frac{f\ (seq_{withA\&B})}{f(seq_{withA}) + f(seq_{withB}) - f\left(seq_{without_{insertion}}\right)}$$

where $f$(seq) corresponds to the predicted activity score for the random backbone sequence, $seq_{withA}$ the predicted activity of the backbone in which motif A was inserted, $seq_{withB}$ the predicted activity of the backbone in which motif B was inserted and $seq_{withA\&B}$ the predicted activity of the backbone in which motif A and B were inserted.

<u>Motif orientation</u>

For each TF pair, 1,000 random 1,000-bp backbone sequences were generated, and the forward consensus sequence of motif A was inserted at the center. The forward consensus sequence of motif B was then inserted at positions corresponding to motif-center distances from −150 to −10 bp and +10 to +150 bp, in 5-bp strides, and cooperativity scores were computed for each position. The same procedure was repeated using the reverse-complement sequence of motif B. The orientation effect was defined as the absolute difference between the median cooperativity scores obtained for the forward and reverse-complement orientations at each motif-center distance. A distance was considered to show a significant orientation effect if both the FDR-corrected Mann–Whitney U test q value was $\leq 0.05$ and the orientation effect was $\geq 0.2$ (SciPy v1.10.1).

<u>Motif distance</u>

Motif insertion and cooperative score calculation were performed as in the previous section (orientation analysis). Subsequently, cooperativity scores obtained for the forward and reverse-complement orientations of TF B were combined, and the median cooperativity score at each absolute distance was computed. Cooperativity scores were considered significant if they differed from the baseline value of 1.0 by at least 0.2 and had an FDR-corrected Wilcoxon signed-rank test q value $\leq 0.05$ (SciPy v1.10.1). For motif pairs showing significant cooperativity at any distance, median cooperativity scores were z-scored across distances, and the resulting cooperativity profiles were clustered using k-means (k = 5; SciPy v1.10.1). For each cluster, the average profile was computed using the cooperativity scores of all the motif pairs assigned to it (available in Supplementary Table 3).

<u>Flanking positions</u>

First, we identified genomic regions in the mm10 genome that were included in the test set of the chromatin accessibility model and matched each motif consensus sequence, with N positions

allowed to match any nucleotide. We then computed contribution scores for each region using DeepSHAP, based on the DeepLIFT implementation in tangermeme.deep_lift_shap (tangermeme v0.4.4). For each motif instance, the mean contribution score across the consensus sequence was calculated, and motif instances were ranked accordingly. We then aggregated the contribution scores at positions −50, −30, −10, −6, −3, −1, +1, +3, +6, +10, +30, and +50 bp from the motif edge in the top 1% and bottom 1% of ranked motif instances. At each flanking position, Welch's t-test was performed to compare the two groups (SciPy v1.10.1), and positions with $p < 0.05$ were considered significant.

Flanking nucleotides

Similar to the previous section, we identified genomic regions containing motif instances. We then tabulated nucleotide composition at positions −5 to −1 and +1 to +5 relative to the motif edges. Motif instances were ranked by the contribution score across the consensus sequence, and flanking nucleotides were visualized as a heatmap. In addition, for each nucleotide at each flanking position, consensus sequence contribution scores were visualized as box plots to assess nucleotide preferences in the flanking regions. Statistical significance among the four nucleotides was evaluated using ANOVA (SciPy v1.10.1). A position was considered significant if $p < 0.001$ and the difference between the highest and lowest median core motif contribution scores exceeded 0.3 times the standard deviation of core motif contribution scores across all motif instances.

## Supplementary Methods References

57. Rauluseviciute, I. et al. JASPAR 2024: 20th anniversary of the open-access database of transcription factor binding profiles. Nucleic Acids Res. 52, D174–D182 (2024).
