# Supplementary material for: Predictive design of tissue-specific mammalian enhancers that function in the mouse embryo
Source: Nat Genet. 2026 Aug 25;58(9):2129–33. doi: 10.1038/s41588-026-02729-1 (PMC13553301; doi:10.1038/s41588-026-02729-1)
Supplement: Supplementary file 2 [file 41588_2026_2729_MOESM2_ESM.pdf]

nature portfolio

Corresponding author(s): Alexander Stark

Last updated by author(s): Jul 6, 2026

Reporting Summary

Nature Portfolio wishes to improve the reproducibility of the work that we publish. This form provides structure for consistency and transparency in reporting. For further information on Nature Portfolio policies, see our Editorial Policies and the Editorial Policy Checklist.

Statistics

For all statistical analyses, confirm that the following items are present in the figure legend, table legend, main text, or Methods section.

n/a	Confirmed	
☐	☒	The exact sample size (*n*) for each experimental group/condition, given as a discrete number and unit of measurement
☐	☒	A statement on whether measurements were taken from distinct samples or whether the same sample was measured repeatedly
☐	☒	The statistical test(s) used AND whether they are one- or two-sided<br>*Only common tests should be described solely by name; describe more complex techniques in the Methods section.*
☒	☐	A description of all covariates tested
☐	☒	A description of any assumptions or corrections, such as tests of normality and adjustment for multiple comparisons
☐	☒	A full description of the statistical parameters including central tendency (e.g. means) or other basic estimates (e.g. regression coefficient) AND variation (e.g. standard deviation) or associated estimates of uncertainty (e.g. confidence intervals)
☐	☒	For null hypothesis testing, the test statistic (e.g. *F*, *t*, *r*) with confidence intervals, effect sizes, degrees of freedom and *P* value noted<br>*Give P values as exact values whenever suitable.*
☒	☐	For Bayesian analysis, information on the choice of priors and Markov chain Monte Carlo settings
☒	☐	For hierarchical and complex designs, identification of the appropriate level for tests and full reporting of outcomes
☐	☒	Estimates of effect sizes (e.g. Cohen's *d*, Pearson's *r*), indicating how they were calculated

Our web collection on statistics for biologists contains articles on many of the points above.

Software and code

Policy information about availability of computer code

Data collection	NA
Data analysis	The code used to train the models, make predictions, generate nucleotide contribution tracks and design new enhancers is available on GitHub (https://github.com/Shenzhi-Chen/DeepSTARR-Mouse). The weights and architecture of the final pre-trained sequence-to-accessibility and sequence-to-activity models are available at https://huggingface.co/Shenzhi-Chen/DeepSTARR-Mouse.<br><br>Biological replicate BAM files were merged using samtools merge (v1.9) Peak calling: MACS2 version 2.2.5<br><br>The models were implemented and trained in python (v3.7.15) using Keras (https://keras.io/, v2.4.3) from TensorFlow (v2.4.1) using the Adam optimizer. We used DeepExplainer (the DeepSHAP implementation of DeepLIFT; updated code at https://github.com/AvantiShri/shap/blob/master/shap/explainers/deep/deep_tf.py) to compute nucleotide contribution scores. De novo motif discovery was performed using TF-MoDISco (modisco-lite v2.4.0). Kernel weights were extracted from the models, and sequence patterns were matched to kernels using Tomtom (v5.5.9) implemented in memelite (v0.2.0). Sequence design is optimized by Ledidi (v2.1.0).<br><br>Designed sequences with a significant match to either the mouse or the human genome were identified using Blastn (via NIH NCBI Blast https://blast.ncbi.nlm.nih.gov/Blast.cgi) All statistical analyses and plots were done in R (v4.4.1). The following R packages were used for downstream analyses: motifmatchr (v1.26.0), hclust (v4.4.1), org.Mm.eg.db (v3.19.1) and AUCell (v1.26.0). The code used for downstream analyses is available on GitHub (https://github.com/vloubiere/chen_loubiere_2025_git).

For manuscripts utilizing custom algorithms or software that are central to the research but not yet described in published literature, software must be made available to editors and reviewers. We strongly encourage code deposition in a community repository (e.g. GitHub). See the Nature Portfolio guidelines for submitting code & software for further information.

Data

Policy information about availability of data

All manuscripts must include a data availability statement. This statement should provide the following information, where applicable:

- Accession codes, unique identifiers, or web links for publicly available datasets
- A description of any restrictions on data availability
- For clinical datasets or third party data, please ensure that the statement adheres to our policy

The training dataset, including all ATAC-seq data from Gorkin et al., Nature 2020, is available through the ENCODE Data Coordination Center (DCC) website using the experiment IDs listed in Supplementary Table 5. In vivo enhancer activity data are publicly available from the VISTA Enhancer Browser (Kosicki et al., NAR 2025). The CTCF ChIP-seq dataset was obtained from Yu et al., NSMB 2025, and the H3K27ac and H3K4me1 datasets were obtained from Sethi et al., Nat. Methods 2020. The data used to train and evaluate the models are available at the Hugging Face repository, DeepSTARR-Mouse-dataset. The transcription factor motif database is available from the Vierstra motif clustering resource. Mouse single-cell RNA-seq data were retrieved from GSE119945.

Research involving human participants, their data, or biological material

Policy information about studies with human participants or human data. See also policy information about sex, gender (identity/presentation), and sexual orientation and race, ethnicity and racism.

Reporting on sex and gender	This study did not involve human participants.
Reporting on race, ethnicity, or other socially relevant groupings	NA
Population characteristics	NA
Recruitment	NA
Ethics oversight	NA

Note that full information on the approval of the study protocol must also be provided in the manuscript.

Field-specific reporting

Please select the one below that is the best fit for your research. If you are not sure, read the appropriate sections before making your selection.

☒ Life sciences ☐ Behavioural & social sciences ☐ Ecological, evolutionary & environmental sciences

For a reference copy of the document with all sections, see nature.com/documents/nr-reporting-summary-flat.pdf

Life sciences study design

All studies must disclose on these points even when the disclosure is negative.

Sample size	The sample size is not applicable to the machine learning models presented in this work. No prior analyses were used to determine the sample size before the experiment.
Data exclusions	Any embryos that were not at the correct developmental stage were excluded from data collection.
Replication	The deep learning models were trained and evaluated in two independent runs using cross-validation, yielding consistent performance across folds and runs. Between 3 and 15 transgenic embryos were collected for each construct. In all cases, the majority of embryos showed a reproducible staining pattern.
Randomization	Not relevant because the samples were not grouped.
Blinding	Investigators were blinded to animals' genotypes during embryo collection and processing.

Reporting for specific materials, systems and methods

We require information from authors about some types of materials, experimental systems and methods used in many studies. Here, indicate whether each material, system or method listed is relevant to your study. If you are not sure if a list item applies to your research, read the appropriate section before selecting a response.

Materials & experimental systems

n/a	Involved in the study
☒	☐ Antibodies
☒	☐ Eukaryotic cell lines
☒	☐ Palaeontology and archaeology
☐	☒ Animals and other organisms
☒	☐ Clinical data
☒	☐ Dual use research of concern
☒	☐ Plants

Methods

n/a	Involved in the study
☒	☐ ChIP-seq
☒	☐ Flow cytometry
☒	☐ MRI-based neuroimaging

Animals and other research organisms

Policy information about studies involving animals; ARRIVE guidelines recommended for reporting animal research, and Sex and Gender in Research

Laboratory animals	Laboratory mice (Mus musculus) were used for in vivo validation. FVB embryos were used for pronuclear injection and targeted integration at the H11 locus, and FVB and CD-1 strains were maintained under standard housing conditions (19–23 °C, 40–60% humidity) with food and water ad libitum on a reversed 12-h dark–light cycle. Embryos were collected at E11.5.
Wild animals	No wild animals were used
Reporting on sex	Embryonic sex was not determined at E11.5; therefore, both sexes were presumed to be included. Sex was not included as a variable in the study design and no sex-specific analyses were performed.
Field-collected samples	No field-collected animals were used
Ethics oversight	All animal procedures were conducted in accordance with NIH guidelines and were approved by the Institutional Animal Care and Use Committee (IACUC) at the University of California, Irvine, under protocol AUP-23-005.

Note that full information on the approval of the study protocol must also be provided in the manuscript.

Plants

Seed stocks	*Report on the source of all seed stocks or other plant material used. If applicable, state the seed stock centre and catalogue number. If plant specimens were collected from the field, describe the collection location, date and sampling procedures.*
Novel plant genotypes	*Describe the methods by which all novel plant genotypes were produced. This includes those generated by transgenic approaches, gene editing, chemical/radiation-based mutagenesis and hybridization. For transgenic lines, describe the transformation method, the number of independent lines analyzed and the generation upon which experiments were performed. For gene-edited lines, describe the editor used, the endogenous sequence targeted for editing, the targeting guide RNA sequence (if applicable) and how the editor was applied.*
Authentication	*Describe any authentication procedures for each seed stock used or novel genotype generated. Describe any experiments used to assess the effect of a mutation and, where applicable, how potential secondary effects (e.g. second site T-DNA insertions, mosiacism, off-target gene editing) were examined.*
