# Supplementary material for: Predictive design of tissue-specific mammalian enhancers that function in the mouse embryo
Source: Nat Genet. 2026 Aug 25;58(9):2129–33. doi: 10.1038/s41588-026-02729-1 (PMC13553301; doi:10.1038/s41588-026-02729-1)
Supplement: Supplementary file 3 [file 41588_2026_2729_MOESM3_ESM.pdf]

nature portfolio

# Peer Review File

## Predictive design of tissue-specific mammalian enhancers that function in vivo in the mouse embryo

Corresponding Author: Dr Alexander Stark

This manuscript has been previously reviewed at another journal. This document only contains information relating to versions considered at Nature Genetics.

Version 0:

Decision Letter:

26th Feb 2026

Dear Alex,

Your Letter, entitled "Predictive design of tissue-specific mammalian enhancers that function in vivo in the mouse embryo", has now been seen by 3 referees. You will see from their comments below that while they find your work of interest, some important concerns are raised, especially by reviewer #1. We are interested in the possibility of publishing your study in Nature Genetics, but would like to consider your response to these concerns in the form of a revised manuscript before we make a final decision on publication.

We therefore invite you to revise your manuscript taking into account all reviewer comments. Please highlight all changes in the manuscript text file. At this stage we will need you to upload a copy of the manuscript in MS Word .docx or similar editable format.

We are committed to providing a fair and constructive peer-review process. Do not hesitate to contact me if there are specific requests from the reviewers that you believe are technically impossible or unlikely to yield a meaningful outcome. I would be happy to discuss the reviewers' points in detail to help streamline the revision process.

When revising your manuscript:

*1) Include a “Response to referees” document detailing, point-by-point, how you addressed each referee comment. If no action was taken to address a point, you must provide a compelling argument. This response will be sent back to the referees along with the revised manuscript.

*2) If you have not done so already please begin to revise your manuscript so that it conforms to our Letter format instructions, available
<a href="http://www.nature.com/ng/authors/article_types/index.html">here</a>.
Refer also to any guidelines provided in this letter.

*3) Include a revised version of any required Reporting Summary: https://www.nature.com/documents/nr-reporting-summary.pdf
It will be available to referees (and, potentially, statisticians) to aid in their evaluation if the manuscript goes back for peer review.
A revised checklist is essential for re-review of the paper.

Please be aware of our <a href="https://www.nature.com/nature-research/editorial-policies/image-integrity">guidelines on digital image standards.</a>

EXTENDED DATA FIGURES

When re-submitting your manuscript, please ensure that any supplementary figures and tables that are crucial to the

manuscript's conclusions are converted into Extended Data figures and tables to increase visibility of these data. Extended Data figures and tables are online-only (present in the online PDF and full-text HTML versions of the paper), peer-reviewed display items that provide essential background to the article but are not included in the main article due to space constraints. A maximum of ten Extended Data display items (figures and tables) is permitted.

Please use the link below to submit your revised manuscript and related files:

Link Redacted

**Note:** This URL links to your confidential home page and associated information about manuscripts you may have submitted, or that you are reviewing for us. If you wish to forward this email to co-authors, please delete the link to your homepage.

We hope to receive your revised manuscript within 3 months. If you cannot send it within this time, please let us know.

Please do not hesitate to contact me if you have any questions or would like to discuss these revisions further.

Nature Genetics is committed to improving transparency in authorship. As part of our efforts in this direction, we are now requesting that all authors identified as 'corresponding author' on published papers create and link their Open Researcher and Contributor Identifier (ORCID) with their account on the Manuscript Tracking System (MTS), prior to acceptance. ORCID helps the scientific community achieve unambiguous attribution of all scholarly contributions. You can create and link your ORCID from the home page of the MTS by clicking on 'Modify my Springer Nature account'. For more information please visit please visit <a href="http://www.springernature.com/orcid">www.springernature.com/orcid</a>.

We look forward to seeing the revised manuscript and thank you for the opportunity to review your work.

Sincerely,

Tiago

Tiago Faial, PhD
Chief Editor
Nature Genetics
https://orcid.org/0000-0003-0864-1200

Reviewers' Comments:

Reviewer #1 (Remarks to the Author):

Overall Assessment

The manuscript demonstrates the ability to design de novo enhancers that function in reporter assays within specific tissues in mammalian embryos. In the present work, the in silico design uses a compact convolutional neural network (CNN) architecture combined with transfer learning. The main result is that a two-stage training strategy (ATAC-seq pretraining followed by VISTA-based fine-tuning) is superior to using either ATAC-seq alone or VISTA alone. The authors test 15 de novo designed enhancers in reporter assays in mouse embryos. This work follows a prior publication from the same lab performed in Drosophila embryos (de Almeida et al., Nature 2024) using what seems to be an essentially identical conceptual framework. To my knowledge the present study is the first such demonstration for the design of synthetic enhancers that can function in a tissue-specific manner within mouse embryos, and this is the main novelty beyond their prior work in Drosophila embryos. While I think this work has strong potential, my main overarching concern is the risk of over-fitting to a small set of known enhancers in the VISTA data set and limitations in new biological insight. The reliance on the VISTA data set also precludes most cell / tissue types that lack such existing enhancer reporter assay data sets. The authors do not seem to claim any new biological knowledge learned by us humans, but there may be potential to gain new biological insights.

Major Comments

1. The VISTA data set is quite small and there is risk of overfitting to that set of known enhancers. The degree of similarity of the designed elements to the VISTA sequences active in the same tissue should be more carefully analyzed. The authors use Word size = 11bp to seed BLAST alignments to conclude that these synthetic enhancers do not align to the genome but such a simple threshold may not give a complete picture. The authors should more carefully quantify the similarity using measures such as Levenshtein distance.
2. Related to #1 -- could the designed elements exhibit synthetic qualities that do not exist in the VISTA data set? This would be a clear functional demonstration that the model has learned some biological principles rather than making minor

modifications to what exists in VISTA. Some options (not all of which need to be performed) may be to:
a) design and test synthetic enhancers to function in two distantly related tissues where none of the VISTA enhancers are simultaneously active
b) design and test enhancers that are substantially smaller than those in the VISTA data set while still achieving comparable activity and tissue specificity, and could be useful for gene therapy vectors in the future
c) design and test enhancers that are substantially stronger in activity than the natural enhancers in the same tissue in the VISTA data set
3. Please analyze/discuss the convolutional filters that may show high contribution but do not match any known TF motifs. Are there any previously unknown motifs that contribute strongly to the prediction score?
4. The manuscript currently does not provide any analysis of potential higher-order TF motif grammar (spacing, orientation, combinations, etc) within the 1kb DNA element that may have been learned by their model. One way to do this may be to analyze what may have been learned by each filter of the CNN. This is where the paper has some potential to provide new insights into biology, especially if any findings related to higher-order TF motif grammar can be functionally tested experimentally.
5. A key limitation of the current framework is its dependence on fine-tuning using the VISTA enhancer data base, which is not an approach that is readily available for most biological contexts. For the approach to be broadly useful to the community, some effort to substitute enhancer reporter assays with more readily scalable data is needed and would demonstrate a clear methodological advance. Even partial exploration of this direction would strengthen the methodological novelty and generalizability of this work. Some options that could be tested include:
a. Using master regulator inference from single-cell or bulk RNA-seq data in place of enhancer reporter assay as training data for fine-tuning in place of VISTA.
b. Using additional active enhancer features (e.g. H3K27Ac and enhancer RNA transcription from nascent RNA sequencing) to identify sequences for fine-tuning in place of VISTA.

Minor Comments

1. Motif reweighting analysis. In terms of how learning results in biologically coherent motif reweighting, it would be helpful to provide additional detail on how motif importance scores were derived and whether these reweighting patterns are robust across different random seeds or training splits.
2. Precision vs. recall trade-off. The prioritization of precision (PPV) over recall is well-justified for the low-throughput in vivo validation context. The authors should still state the recall values achieved and discuss how this trade-off might shift if the approach were to be scaled to higher-throughput validation settings.
3. It would be helpful for the paper to discuss whether the model learns any Tn5 sequence-related technical bias in ATAC-seq and whether accounting for that may or may not be useful.

Reviewer #2 (Remarks to the Author):

Remark to the authors:

Recent advances in genomics and machine learning models allow the design of synthetic regulatory elements. This will not only enlighten fundamental principles of transcriptional activation but also holds great promise for future clinical applications. The work by Chen et al. introduces a strategy for designing mammalian tissue-specific enhancers, which they show to be functional in vivo in a LacZ reporter assay in mouse embryos. Somewhat similar approaches have already proven to be efficient both in mammalian cells and in vivo, in Drosophila. One of these studies was published in 2023 by the same lab. They have now applied a similar methodology using mouse embryo ATAC-seq data combined with the powerful resource of the VISTA enhancer database. This is the first time that such an approach has been used in mammalian tissues in vivo, and the results are very convincing. I thus believe that this work fits the scope of Nature Genetics. However, before publication is possible, I would need to see some clarifications on their approach and the results that I have listed below.

1- Is there any specific reason why the authors chose chromosome 18 (and not another chromosome) not to be part of the training? Any size, known enhancers, … justification? Could the authors please add a sentence justifying the reason or absence of reason, either in the main text or the methods?

2- Extended Data Figure 1a: I am not sure I understand what the goal is to show the % of ATAC peaks overlapping TSS. Are those peaks discarded of the analysis to make sure the model learn with enhancers and not promoters? Could the author please clarify this?

3- Figure 1b: Could the authors add a scale for the observed ATAC peaks? It would also be informative to have information about what enhancers/gene loci we are looking at.

4- Figure 1c: are those regions all from chr18? Could this be stated somewhere?

5- The model rejected 300,000 randomly generated sequences (line 97). How were those sequences generated? Randomly generated sequences can carry TFBS and there are examples in the literature of random sequences driving expression (work from the Crocker lab, for example). Could the author briefly comment on why all the 300,000 randomly generated sequences are rejected by their model?

6- Extended Data Figure1i-j: Can the authors comment on why the model trained directly on VISTA performs so well on the heart compared to other tissues? Also, in j, why is the model trained directly on VISTA not plotted for the midbrain? From i, it seems that it is close to 0 for limb but not for midbrain.

7- Figure 2a: could the authors provide the list of all TF motif contributions (either on the figure or better as a supplementary file)?

8- Could the authors provide more information on the results of the de novo design of synthetic enhancers using their model? How many sequences per tissue? What was the threshold for scoring these de novo enhancers as good candidates? How were the 5 tested tissue-specific enhancers chosen in this list? Were they the ones with the highest score? It is impressive that all 15 tested enhancers lead to somewhat specific LacZ expression, but I wonder if this is because the author chose the 5 “best” ones. From Supplementary Table 1, it seems that the predicted tissue-specific accessibility is similar for all synthetic enhancers from the same tissues. Do all predicted enhancers have a similar score?

9- LacZ staining for testing of the synthetic enhancer.
- The methods for the LacZ staining are missing from the Material/Methods section. Particularly, it would be important to know how long the embryos have been stained for (less than 1h, a couple of h or more than 24h?). The better an enhancer works, the quicker the LacZ staining should appear. Were all 15 enhancers stained for the same time?
- Also, how was this compared to a strong endogenous enhancer (for example, the tested VISTA enhancers)? LacZ staining is not a quantifiable assay, but this can be done by RT-qPCR. If possible, could the expression of LacZ be quantitatively compared to an endogenous enhancer?
- On the extended Data Figure, “single” and “tandem” appear. Could the author clarify why certain insertions are in tandem?
- The embryo shown in the main figure for the heart enhancer #5 is not an E11.5. Could the author use image #2 (from the Extended Data Figure) in the main figure instead? Also, this enhancer #5 clearly shows lower expression than the four others. This should be mentioned.
- The embryos for the CNS enhancer #4 are not all looking good. Any specific reason for that?
- The authors called 9 embryos out of 14 positively stained as heart-specific expression for heart enhancer #1. However, I am not convinced about embryos 8 and 9. The expression is ubiquitous, so I would suggest calling only 7/14 positive embryos.

10- Would it be possible to show the functionality of the synthetic enhancer in an endogenous context? For example, knock in one of the synthetic limb enhancers at the Shh locus in a ZRS mutant (maybe add REX?). Would this rescue any expression of Shh? In my opinion, this will be very relevant, and I would love to see something like that, but I am aware that such in vivo experiments can be time-consuming.

11- Many sequences defined as “enhancers” with the sequence to accessibility model were not shown as enhancers with the VISTA sequence-to-activity model. So, what differentiates the accessible region that can drive expression to the one which cannot? Is it only TFBS? I am not asking for any further experiment here, but maybe some discussion about that.

12- How similar in sequence are the 5 designed enhancers for a specific tissue? From Fig. 2b, it seems that all enhancers have TFBS motifs for the mentioned TF, but what could drive the differences in the LacZ expression pattern? Do they all have the same number and order of TFBS and similar spacing between those motifs?

Minor comments:
1- Since the submission of this paper, a new work was published using MPRA-based deep learning models to design synthetic human promoter sequences (https://doi.org/10.1038/s41586-025-10093-z). Citing this work might be relevant.
2- Please define the acronym PCC (Pearson Correlation Coefficient) in the main text. It currently appears only in the methods.
3- Extended data 1b is cited before 1a in the text.

Reviewer #3 (Remarks to the Author):

Chen et al. present an approach to design mammalian tissue-specific enhancer sequences using a deep learning model. The approach first trains a relatively simple CNN to predict ATAC-seq signals across three tissue types (heart, limb, midbrain). The model is then fine-tuned using a small set of experimentally validated enhancer sequences from the VISTA enhancer browser. The Ledidi model is then used to generate synthetic enhancer sequences from the trained CNN and several generated sequences were tested for enhancer activity in mouse embryos. While other studies have demonstrated the feasibility of using deep learning models to generate synthetic enhancer sequences, the importance of this study lies in the simple training procedure (i.e., relying first on an ATAC-seq training objective). Previous studies have relied on MPRA training data, so the feasibility of training with accessibility data greatly broadens the number of cell/tissue types that might be targeted by enhancer design techniques. This is also, to my knowledge, the first study that characterizes enhancer activity of designed sequences in mouse embryos, demonstrating the success of the approach.

Overall, this is a compact and well-executed study. I only have two minor comments for clarification:

1. The methods state that "seed sequences were then optimized using both the sequence-to-accessibility and sequence-to-activity models...". This should be clarified. Were distinct sequences designed using each model? If so, which models

generated each of the tested sequences? Or, alternatively, was there some form of joint optimization using both models?
2. Figure 2a shows how motif contribution scores change before and after transfer learning. It would be more informative to label all rows in this figure.

Version 1:

Decision Letter:

Our ref: NG-LE71722R

1st May 2026

Dear Alex,

Thank you for submitting your revised manuscript entitled "Predictive design of tissue-specific mammalian enhancers that function in vivo in the mouse embryo" (NG-LE71722R). It has now been seen by the original referees and their comments are below. The reviewers find that the paper has improved in revision, and therefore we'll be happy in principle to publish it in Nature Genetics, pending minor revisions to satisfy the referees' final requests and to comply with our editorial and formatting guidelines.

We are now performing detailed checks on your paper and will send you a checklist detailing our editorial and formatting requirements soon. Please do not upload the final materials and make any revisions until you receive this additional information from us.

Thank you again for your interest in Nature Genetics. Please do not hesitate to contact me if you have any questions.

Congratulations!

Sincerely,

Tiago

Tiago Faial, PhD
Chief Editor
Nature Genetics
https://orcid.org/0000-0003-0864-1200

Reviewer #1 (Remarks to the Author):

The authors addressed the concern of similarity of the designed enhancers to VISTA sequences adequately. The authors also added additional analyses using chromatin features for fine-tuning in place of VISTA, which substantially widens the applicability of their approach to other biological systems. The authors also added analyses of potential novel and higher-order motif patterns which may inform future functional studies beyond the scope of this manuscript. Altogether, the authors have adequately addressed the concerns I raised during first round of review. In my view, this manuscript advances the field of genetics in a substantial manner and is suitable for publication.

Reviewer #1 (Remarks on code availability):

Please ensure that the code that uses chromatin features as a replacement for VISTA for fine-tuning are also shared.

Reviewer #2 (Remarks to the Author):

The authors have answered most of my questions. I am overall satisfied by their answers and additional analysis and data. However, before publication, I am asking for some minor corrections:

1) On Figure 2b, #enhancer 1 is still indicated as having 9/14 embryos with similar staining while I asked the reviewers to correct that. See previous comment point 9 and their answer here:
“- The authors called 9 embryos out of 14 positively stained as heart-specific expression for heart enhancer #1. However, I am not convinced about embryos 8 and 9. The expression is ubiquitous, so I would suggest calling only 7/14 positive embryos.
We thank the reviewer for this careful assessment. We agree that embryos 8 and 9 show more diffuse staining and have therefore revised the scoring to 7/14 embryos with clear heart-specific expression. This has been updated in the figure and

corresponding text.”

2) The authors have added Figure 3 showing the validation of forebrain and midbrain-specific enhancers. In the current revised version of the manuscript, the text describing this figure is in the Discussion part. This should be move to the results. As indicated by the authors in the manuscript (“Together, these results demonstrate that …” line 224-225), this is a result and not a discussion point.

3) This new Figure 3 shows embryos with GFP and/ or mCherry expression. When testing MB1 and FB1, the authors are showing only one embryo and indicate that this staining was observed for 1 out of 1 embryo. These are not statistically relevant results, and I would like to see replicates for that construct and at least 3 embryos showing similar stainings. It is particularly important as we can observe from the current Extended data 8c that the staining is highly variable between different embryos (for FB2). I believe that this needs to be corrected before publication.

Reviewer #3 (Remarks to the Author):

I had only minor comments on the first manuscript submission. The revised manuscript is even further improved. I have no further comments at this time.

**Open Access** This Peer Review File is licensed under a Creative Commons Attribution 4.0 International License, which permits use, sharing, adaptation, distribution and reproduction in any medium or format, as long as you give appropriate credit to the original author(s) and the source, provide a link to the Creative Commons license, and indicate if changes were made.
In cases where reviewers are anonymous, credit should be given to 'Anonymous Referee' and the source.
The images or other third party material in this Peer Review File are included in the article’s Creative Commons license, unless indicated otherwise in a credit line to the material. If material is not included in the article’s Creative Commons license and your intended use is not permitted by statutory regulation or exceeds the permitted use, you will need to obtain permission directly from the copyright holder.
To view a copy of this license, visit https://creativecommons.org/licenses/by/4.0/

## Summary of the changes introduced in the revised manuscript

We thank all three reviewers and the editor for their feedback, which prompted us to perform additional benchmarking, expand our computational analysis of motifs and motif grammar, and extend our transfer-learning approach. All new additions to the revised manuscript have been highlighted in blue in the text and in the methods section. In particular, the revised manuscript now includes:

- The demonstration that the 15 synthetic enhancers do not resemble VISTA enhancers, ruling out overfitting
- An in-depth computational analysis of motifs and motif grammar
- Evidence that cross-validation, transfer learning, and benchmarking are robust and reproducible
- New in vivo validated synthetic enhancers targeting distinct CNS subregions (forebrain and midbrain), demonstrating the framework's capacity for fine-grained regional specificity (introduced in the new main Figure 3)
- An extension of our transfer-learning framework to settings without validated enhancers. This substantially extends the scope and applicability of our framework to cell types and tissues not covered by VISTA, thereby broadening its use in contexts where enhancer activity data are unavailable

Together, the revised manuscript contains **3 main figures** (up from 2):

1. Deep learning-based prediction and design of tissue-specific mammalian enhancers
2. In vivo validation of tissue-specific synthetic enhancers in mouse embryos
3. Design and validation of forebrain and midbrain-specific synthetic enhancers

**8 extended data figures** (up from 3), plus supplementary information

1. Sequence-to-accessibility model performance assessment
2. Sequence-to-activity model training and performance assessment
3. Reproducibility and analyses of motif-reweighting during transfer learning
4. Sequence comparisons and features of synthetic enhancers
5. LacZ stained E11.5 embryos for all tested enhancers
6. Transfer learning using inferred enhancer-activity labels
7. De novo motifs and higher-order motif-syntax rules
8. Performance of CNS-subregion-specific models and in vivo validation

SI: Performance of sequence-to-accessibility models on chr18 versus others

And **9 supplementary tables** (up from 6):

1. Complete list of motifs and scores associated to Fig. 2a
2. Designed synthetic enhancers selected for in vivo validation
3. Higher-order motif syntax rules
4. Midbrain and forebrain-specific synthetic enhancers selected for in vivo validation
5. Publicly available ATAC-seq BAM files used in this study
6. Full list of annotated ATAC-Seq peaks
7. Full list of VISTA Enhancer Browser sequences
8. Number of sequences used for sequence-to-accessibility models
9. Number of sequences used for sequence-to-activity models.

**Reviewer #1 (Remarks to the Author):**

Overall Assessment

The manuscript demonstrates the ability to design de novo enhancers that function

in reporter assays within specific tissues in mammalian embryos. In the present work, the in silico design uses a compact convolutional neural network (CNN) architecture combined with transfer learning. The main result is that a two-stage training strategy (ATAC-seq pretraining followed by VISTA-based fine-tuning) is superior to using either ATAC-seq alone or VISTA alone. The authors test 15 de novo designed enhancers in reporter assays in mouse embryos. This work follows a prior publication from the same lab performed in Drosophila embryos (de Almeida et al., Nature 2024) using what seems to be an essentially identical conceptual framework. To my knowledge the present study is the first such demonstration for the design of synthetic enhancers that can function in a tissue-specific manner within mouse embryos, and this is the main novelty beyond their prior work in Drosophila embryos. While I think this work has strong potential, my main overarching concern is the risk of over-fitting to a small set of known enhancers in the VISTA data set and limitations in new biological insight. The reliance on the VISTA data set also precludes most cell / tissue types that lack such existing enhancer reporter assay data sets. The authors do not seem to claim any new biological knowledge learned by us humans, but there may be potential to gain new biological insights.

We thank the reviewer for recognizing that our study is the first to design synthetic enhancers that function in a tissue-specific manner in mouse embryos and represents a key advance beyond prior work in Drosophila. We are also grateful for raising important points regarding potential overfitting, the applicability of the framework to settings not covered or under-represented in the VISTA enhancer browser database, and opportunities to extract additional biological insights. In the following, we address all comments point-by-point.

Major Comments

1. The VISTA data set is quite small and there is risk of overfitting to that set of known enhancers. The degree of similarity of the designed elements to the VISTA sequences active in the same tissue should be more carefully analyzed. The authors use Word size = 11bp to seed BLAST alignments to conclude that these synthetic enhancers do not align to the genome but such a simple threshold may not give a complete picture. The authors should more carefully quantify the similarity using measures such as Levenshtein distance.

We agree with the reviewer that the relatively small size of the VISTA dataset presents a potential risk of overfitting. In the original manuscript, we mitigated this risk through rigorous cross-validation, the use of the editing method Ledidi with random starting sequences, and by ensuring that designed sequences do not exhibit significant similarity to VISTA enhancers or other genomic sequences using BLAST (word size 11, default settings).

We appreciate the suggestion to further quantify sequence similarity using complementary approaches. We have therefore extended our analysis and show that the designed enhancers are as dissimilar to VISTA enhancers as to randomly generated sequences when assessed using both Levenshtein and Hamming distances (new Extended Data Fig. 4f):

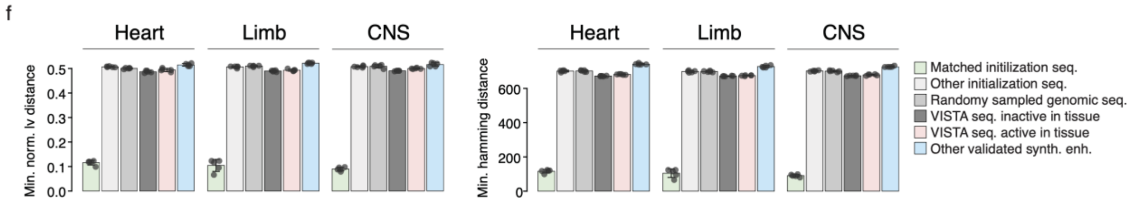


***Extended data Figure 4f:*** *Normalized Levenshtein distances (left) and Hamming distances (right) between validated synthetic enhancers and other sets of sequences (see color legend). In order, these include the matched initialization sequence from which the enhancers were derived (1 per enhancer), other random initialization sequences (n= 1,200), randomly sampled genomic sequences (n= 1,000), VISTA sequences that are inactive (n= 2,895, 2,873, 2,774) or active (n= 311, 333, 432) in heart, limb and midbrain, respectively; and the other validated synthetic enhancers (n= 15).*

In addition, we performed BLAST with increased sensitivity (word size 7 bp, which seeds alignments on shorter exact matches and is therefore more likely to detect partial or degenerate similarities) and found that none of the 15 synthetic enhancers shows significant similarity to VISTA sequences or to the mouse or human genome, and updated the Methods accordingly (lines 505-509).

Beyond sequence-level similarity, we further analyzed motif content in designed sequences, validated enhancers, and ATAC-seq peaks. As expected, designed enhancers share key tissue-specific TF motifs with VISTA enhancers and ATAC-seq peaks: this is a necessary feature of functional enhancers rather than evidence of overfitting, since the models must learn genuine regulatory grammar to design active sequences. Importantly, however, their overall motif composition remains distinct from that of VISTA enhancers, ATAC-seq peaks, and VISTA inactive sequences, and hierarchical clustering based on full motif content clearly separates the designed enhancers from all these groups, placing them as outgroups in the dendrogram. We infer that the models have not memorized VISTA sequences but have instead learned generalizable sequence features (see new Extended Data Fig. 4d):

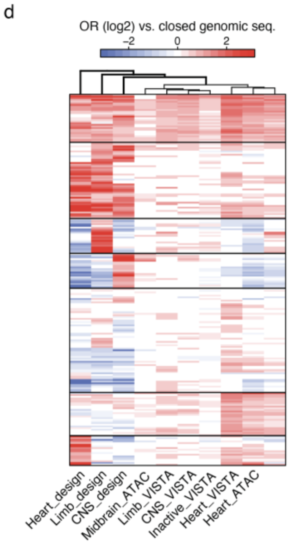


***Extended Data Fig. 4d:*** *Hierarchical clustering (euclidean distances, complete linkage, see dendrogram on top) of designed synthetic enhancers, VISTA enhancers, ATAC-seq peaks, and VISTA inactive control sequences based on their motif enrichments. e- Length distribution of VISTA sequences active in heart, limb or midbrain (x axis labels) versus the lengths of validated enhancers (black) as well as the distance between outermost ledidi edits (red) or important seqlets (zscore>1, in green) are shown.*

In addition, we now provide the motif counts within synthetic enhancers, for the top enriched motifs within each group (see new Extended Data Fig. 4c).

Together, these analyses demonstrate that the designed enhancers are no more similar to VISTA sequences than to random genomic or entirely random sequences, providing strong evidence that the models have learned generalizable regulatory features rather than overfitting to the training data.

2. Related to #1 -- could the designed elements exhibit synthetic qualities that do not exist in the VISTA data set? This would be a clear functional demonstration that the model has learned some biological principles rather than making minor modifications to what exists in VISTA. Some options (not all of which need to be performed) may be to:
a) design and test synthetic enhancers to function in two distantly related tissues where none of the VISTA enhancers are simultaneously active
b) design and test enhancers that are substantially smaller than those in the VISTA data set while still achieving comparable activity and tissue specificity, and could be useful for gene therapy vectors in the future
c) design and test enhancers that are substantially stronger in activity than the natural enhancers in the same tissue in the VISTA data set

We thank the reviewer for these thoughtful suggestions. Indeed, our data strongly suggest that the designed elements exhibit synthetic qualities that do not exist in the VISTA dataset. First, the designed enhancers are no more similar to VISTA sequences than to random sequences across multiple measures, arguing against simple pattern matching (see our response to comment 1). Second, our new motif grammar analyses (new Extended Data Fig. 7 and Supplementary Table 3) reveal that the models have learned generalizable regulatory features consistent with grammar rules we previously identified and experimentally validated in an entirely unrelated system (de Almeida et al, Nature Genetics 2022). Together, these suggest the models have internalized aspects of enhancer syntax rather than reproducing known sequences.

Regarding suggestion (a), all the tissues considered here share at least 14 VISTA enhancers as we now show in Extended Data Fig. 2c, such that combinations of activity patterns that are not in VISTA don’t exist for the three modeled tissues. We therefore decided to test whether we could create enhancers that are specific to either the forebrain or the midbrain subregion of the CNS. This choice is motivated by a key future direction of designing more refined enhancers and because most VISTA enhancers show overlapping activities between different CNS subregions (Extended Data Fig. 2c). The validation of these enhancers using our dual-color reporter system in vivo in mouse embryos is now presented in lines 214-227 and shown as new main Fig. 3:

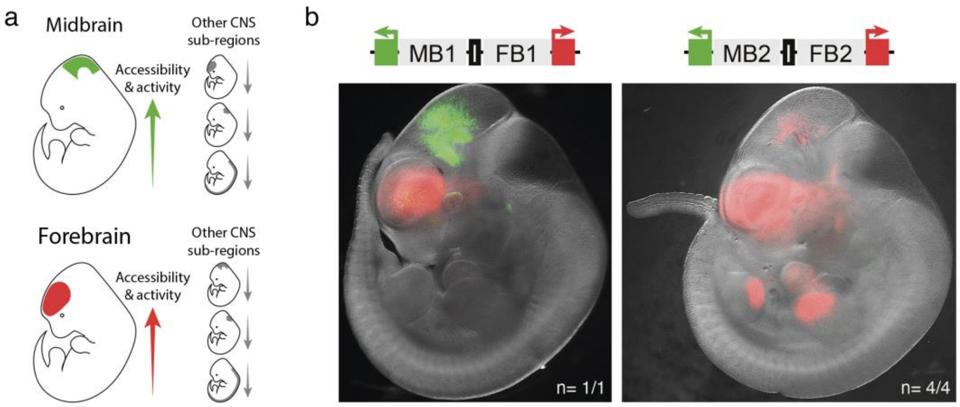


***Figure 3: Design and validation of forebrain and midbrain-specific synthetic enhancers***
*a- Schematic overview of the contrastive approach used to design midbrain-specific (top) and forebrain-specific (bottom) synthetic enhancers. Sequences were designed to have high accessibility and activity scores in the target tissue, and low accessibility and activity scores in the three other CNS sub-regions, which include forebrain, midbrain, hindbrain and neural tube. b- Transgenic E11.5 mouse embryos carrying dual-reporter constructs (top), in which synthetic midbrain (MB) and forebrain (FB) enhancers were placed upstream of GFP (green) and mCherry (red), respectively, and separated by an insulator sequence (black). n indicates the number of positive embryos among all recovered embryos that carry the construct (see Extended Data Figure 8c for all recovered embryos).*

We found that 3 out of 4 enhancers drove activity in their intended subregion, and one forebrain enhancer showed truly forebrain-specific activity. These results demonstrate that the framework can target fine-grained CNS subregional specificity, providing an exciting proof-of-concept for future work towards designing tissue and cell type-specific enhancers (lines 214-227).

Regarding suggestion (b), the designed enhancers (1,001 bp) are already substantially shorter than most VISTA enhancers, as 84% of VISTA enhancers exceed 1 kb in length (average length: 2,250 bp). Moreover, the sequence edits introduced during the design process are typically confined to windows of 658–815 bp, suggesting that compact functional sequence elements are sufficient to drive activity. These sequence-length comparisons are now included in the new Extended Data Fig. 4e:

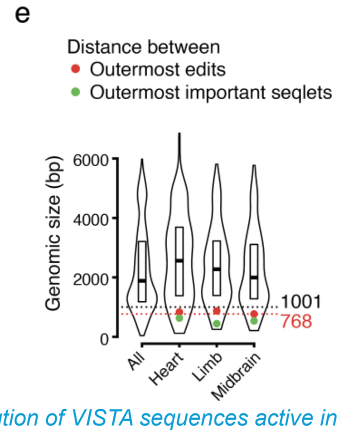


***Extended Data Fig. 4e:*** *Length distribution of VISTA sequences active in heart, limb or midbrain (x axis labels) versus the lengths of validated enhancers (black) and the distance between outermost Ledidi edits (red) or important seqlets (zscore>1, in green).*

The primary goal of the present study is to establish the feasibility of designing tissue-specific enhancers that function in vivo in mammalian embryos. The

remaining suggested experiments are highly interesting extensions that are now enabled by this framework, as we highlight in the revised Discussion (lines 236-241). However, testing these ideas in vivo would require extensive additional experiments in mouse embryos, which would take considerable time and, in our view, are beyond the scope of the current study. We hope to pursue these directions in follow-up work.

3. Please analyze/discuss the convolutional filters that may show high contribution but do not match any known TF motifs. Are there any previously unknown motifs that contribute strongly to the prediction score?
We thank the reviewer for this suggestion, which prompted us to substantially extend our analysis. Using TF-MoDISco, which identifies recurrent high-contribution sequence patterns based on model attributions, we find that the models recover many known TF motifs and motif-like patterns as well as patterns that resemble composite motifs. For example, the limb model learned a previously undescribed motif (CTATTCA) and a composite motif between the LMX1B and TWIST1 motifs (see new Extended Data 4a-b):

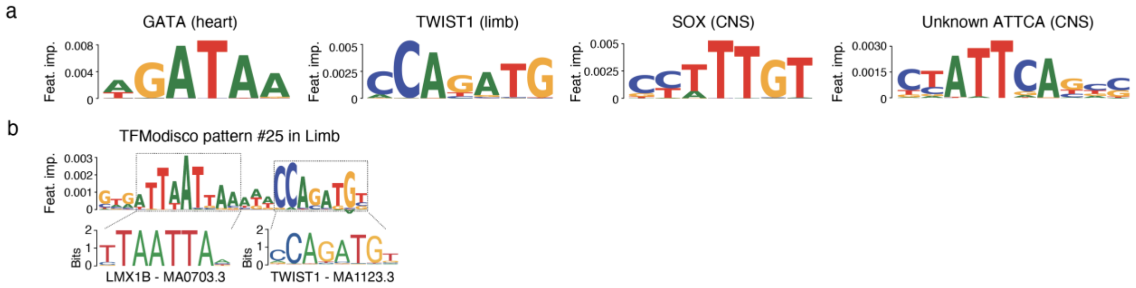


***Extended Data Fig. 7a-b:*** *a- Examples of high-contribution patterns identified by TF-Modisco in heart, limb and CNS sequence-to-accessibility models, with the associated TF when a significant hit was found in the JASPAR motif database. b- Example of a composite LMX1B/TWIST1 motif identified by TF-Modisco.*

In addition, we directly examined the learned convolutional filters and found that several filters capture recognizable TF motifs, consistent with the TF-MoDISco results (see sheets 2-4 in the new Supplementary Table 3).

Given the lack of direct experimental validation and to maintain focus on the main message, we present these analyses briefly in the main text (lines 192-199) and in full detail in the new Extended Data Fig. 7 and the new Supplementary Table 3, including an extensive list of de-novo identified motifs, their annotations, and corresponding convolutional filters.

4. The manuscript currently does not provide any analysis of potential higher-order TF motif grammar (spacing, orientation, combinations, etc) within the 1kb DNA element that may have been learned by their model. One way to do this may be to analyze what may have been learned by each filter of the CNN. This is where the paper has some potential to provide new insights into biology, especially if any findings related to higher-order TF motif grammar can be functionally tested experimentally.

We thank the reviewer for this suggestion and have now analyzed the impact of motif spacing, orientation, combinations, and flanking sequences on predicted enhancer activity. These analyses reveal multiple examples of higher-order regulatory

grammar, including heart-specific strong cooperative interactions between MEF2 motifs and limb-specific interactions between TWIST1 and HOXD9 motifs, as well as weaker but significant interactions between MEF2 and GATA motifs in the heart (new Extended Data Fig. 7d):

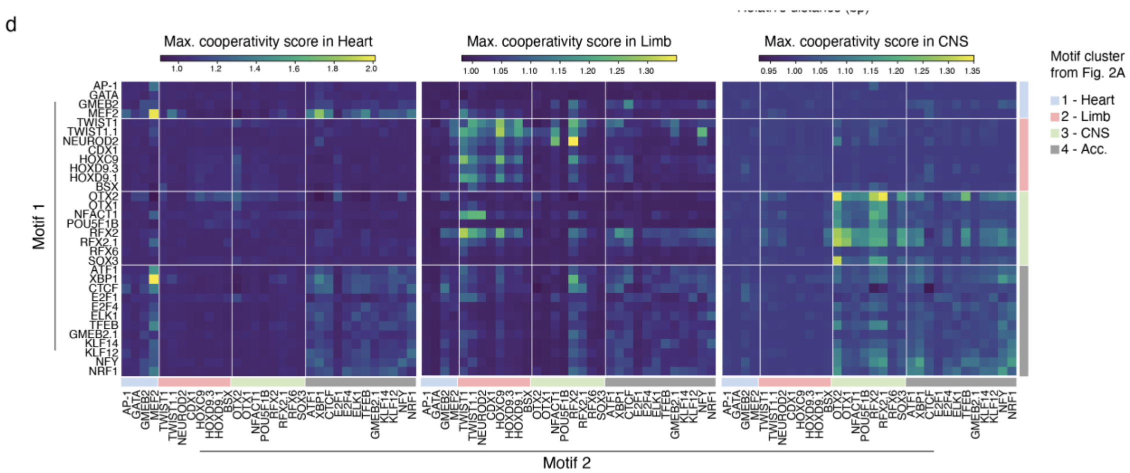


*Extended Data Fig. 7d: Cooperativity between motif pairs. For each pair of motifs from Fig. 2A, the maximum cooperativity score reached at optimal inter-motif distance is shown.*

We also assessed the effects of inter-motif distance on synergy and the contribution of flanking nucleotides to motif activity (new Extended Data Fig. 7e-f):

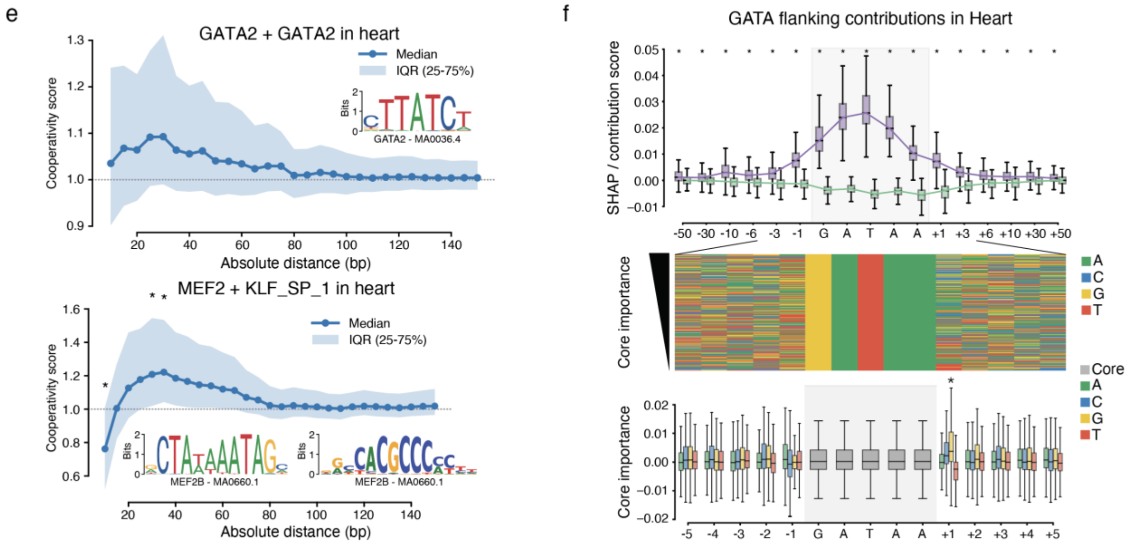


***Extended Data Fig. 7e-f:*** *e- Motif cooperativity at increasing inter-motif distances for a GATA-GATA and a MEF2-KLF motif pair (heart model). f- Impact of flanking nucleotides on GATA motif importance in heart sequence-to-activity model. Shown are attribution scores of the most highly versus the most poorly scoring 10% of GATA-motif instances, irrespective of nucleotide identity (top) and the nucleotide identity next to highly versus poorly scoring GATA-motif instances (bottom). For all patterns identified by TF-Modisco and analyses of higher-order motif syntax, see Supplementary Table 3.*

Notably, these patterns are consistent with grammar rules that we previously characterized and experimentally validated using high-throughput assays in an independent system (de Almeida et al., 2022). Together, these findings suggest that the models capture aspects of regulatory grammar beyond individual motifs.

These findings represent computational predictions derived from the models. Direct experimental validation in heart, limb, or CNS tissues would require many in vivo assays in mouse embryos and is, in our view, beyond the scope of the present study. However, their consistency with grammar rules we previously identified and experimentally validated using high-throughput reporter assays in an entirely independent system (de Almeida et al., 2022) lends them biological credibility and suggests they reflect genuine regulatory logic. Specifically, the GATA motif spacing preferences and flanking nucleotide effects recovered here closely mirror those validated in our prior study, despite the use of different tissues, organisms, and experimental platforms.

We present these analyses as testable predictions and note that their experimental validation in mammalian tissues is a now tractable direction for future work, ideally using a quantitative high-throughput reporter assays (as in de Almeida et al., 2022). We have framed the results accordingly in the revised manuscript (lines 192-204, new Extended Data Fig. 7 and Supplementary Table 3).

5. A key limitation of the current framework is its dependence on fine-tuning using the VISTA enhancer data base, which is not an approach that is readily available for most biological contexts. For the approach to be broadly useful to the community, some effort to substitute enhancer reporter assays with more readily scalable data is needed and would demonstrate a clear methodological advance. Even partial exploration of this direction would strengthen the methodological novelty and generalizability of this work.

Some options that could be tested include:
a. Using master regulator inference from single-cell or bulk RNA-seq data in place of enhancer reporter assay as training data for fine-tuning in place of VISTA.
b. Using additional active enhancer features (e.g. H3K27Ac and enhancer RNA transcription from nascent RNA sequencing) to identify sequences for fine-tuning in place of VISTA.

We thank the reviewer for highlighting the importance of extending our framework to cell types and tissues for which no VISTA enhancers are available. We fully agree that reducing the dependence on enhancer reporter assays would substantially broaden the applicability of our method.

We have now addressed this point by leveraging established features of active enhancers to generate training labels independently of VISTA. Specifically, we tested several strategies based on readily available genomic data, including (1) ATAC-seq peaks distal from promoters and CTCF-bound regions, (2) ATAC-seq peaks marked by H3K27Ac and H3K4me1, and (3) tissue-specific ATAC-seq peaks accessible only in the target tissue. We focused on these established enhancer features as they are broadly available across diverse biological contexts.

We first show that all three approaches enrich for active VISTA enhancers relative to accessibility alone (new Extended Data Fig. 6a), confirming that these features can be used as predictors of enhancers in the absence of direct reporter assay data. We then use these inferred enhancer labels for transfer learning, effectively replacing VISTA enhancers, and evaluate model performance against VISTA as ground truth.

These analyses demonstrate that models trained with inferred positives perform substantially better than sequence-to-accessibility models across all three tissues and, for tissue-specific ATAC-seq peaks, approach the performance of sequence-to-activity models fine-tuned on VISTA data. As expected, models fine-tuned on VISTA data remain the most accurate and robust across the three tissues (new Extended Data Fig. 6b):

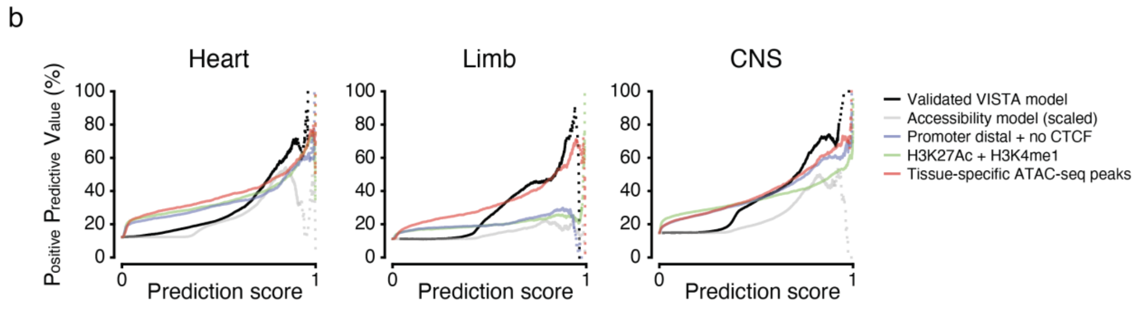


***Extended Data Fig. 6b:*** *PPV of enhancer activity across prediction score thresholds, comparing the validated sequence-to-activity model fine-tuned on VISTA (black) to models trained using inferred enhancer labels predicted based on the enhancer hallmarks defined in panel a (color legend). Scaled sequence-to-accessibility model is shown as a reference (grey).*

These results substantially expand the scope and applicability of our framework, indicating that enhancer design can be extended to biological contexts for which no experimentally validated enhancers are available. We have incorporated these analyses in the revised manuscript as a new results section and Extended Data Figure 6 and discuss their implications for generalizing enhancer design beyond VISTA-supported tissues (lines 153-171 and 208-213).

Minor Comments

1. Motif reweighting analysis. In terms of how learning results in biologically coherent motif reweighting, it would be helpful to provide additional detail on how motif importance scores were derived and whether these reweighting patterns are robust across different random seeds or training splits.

We have now expanded the description of how motif importance scores are derived in the Methods section (lines 512-519) and refer to the new Supplementary Table 1 in the main text (line 115), which contains all the scores per motif and per tissue. In addition, we now compute motif reweighting patterns across all folds and replicates and find that these patterns are highly reproducible. These results are now described in the new Extended Data Fig. 3a:

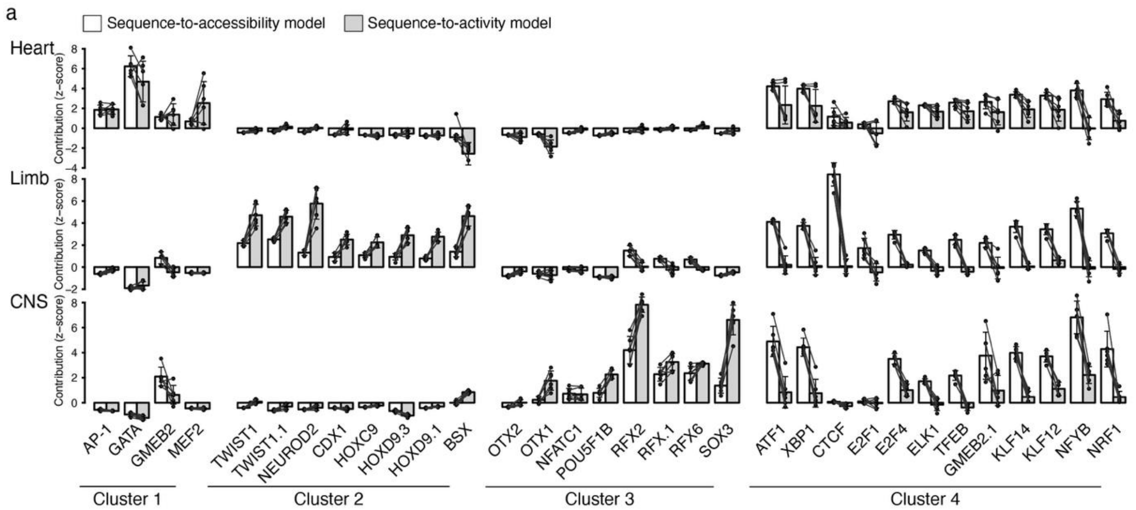


***Extended Data Fig. 3a:*** *Scaled contribution scores of the important motifs shown in Fig. 2a, for each of the three folds and two replicates (n=6 total, black dots) of sequence-to-accessibility (white) and sequence-to-activity (grey) models per tissue (see labels on the left). Bars heights indicate mean values and whiskers the standard deviation.*

2. Precision vs. recall trade-off. The prioritization of precision (PPV) over recall is well-justified for the low-throughput in vivo validation context. The authors should still state the recall values achieved and discuss how this trade-off might shift if the approach were to be scaled to higher-throughput validation settings.

We now provide precision and recall at different cutoffs, plot Precision-Recall curves and evaluate AUCs (new Extended Data fig. 2a-b). We also discuss how precision and recall can be balanced in various settings (lines 91-92 and 236-238).

3. It would be helpful for the paper to discuss whether the model learns any Tn5 sequence-related technical bias in ATAC-seq and whether accounting for that may or may not be useful.

We thank the reviewer for this suggestion. We examined whether Tn5 sequence bias is captured by our models by analyzing the motifs recovered using TF-MoDISco. We did not observe patterns consistent with known Tn5 sequence bias among the motifs identified by either the sequence-to-accessibility or sequence-to-activity models (see new Supplementary Table 3, sheets 2-4). This is consistent with previous work (ChromBPNet; Pampari et al., 2024, bioRxiv; now cited as ref #47), which suggests that Tn5 bias primarily affects nucleotide-resolution profile predictions, but is less relevant for predictions of overall accessibility signal (e.g., peak height), as performed here. We now mention this in the revised manuscript (lines 423-427).

**Reviewer #2 (Remarks to the Author):**
Remark to the authors:
Recent advances in genomics and machine learning models allow the design of synthetic regulatory elements. This will not only enlighten fundamental principles of transcriptional activation but also holds great promise for future clinical applications. The work by Chen et al. introduces a strategy for designing mammalian tissue-specific enhancers, which they show to be functional in vivo in a LacZ reporter assay in mouse embryos. Somewhat similar approaches have already proven to be

efficient both in mammalian cells and in vivo, in Drosophila. One of these studies was published in 2023 by the same lab. They have now applied a similar methodology using mouse embryo ATAC-seq data combined with the powerful resource of the VISTA enhancer database. This is the first time that such an approach has been used in mammalian tissues in vivo, and the results are very convincing. I thus believe that this work fits the scope of Nature Genetics. However, before publication is possible, I would need to see some clarifications on their approach and the results that I have listed below.

We thank the reviewer for the positive and supportive evaluation, and for highlighting that it demonstrates, for the first time, tissue-specific enhancer design in mammalian tissues in vivo. We are also grateful to the reviewer for noting that the results are convincing and within the scope of *Nature Genetics*. We appreciate the reviewer's thoughtful comments and suggestions and address all points in detail below.

1- Is there any specific reason why the authors chose chromosome 18 (and not another chromosome) not to be part of the training? Any size, known enhancers, … justification? Could the authors please add a sentence justifying the reason or absence of reason, either in the main text or the methods?

We thank the reviewer for this question. While chromosome 18 was systematically excluded from training, each fold also included an independent test set of sequences randomly drawn from all chromosomes. The purpose of excluding a single chromosome across all folds was to enable direct comparison of model performance on a consistent set of sequences; the choice of chromosome 18 itself was arbitrary.

We now clarify this in the revised manuscript (lines 73-77) and additionally show that model performance is comparable on test sequences from chromosome 18 and from other chromosomes (new Supplementary Information, Panel a):

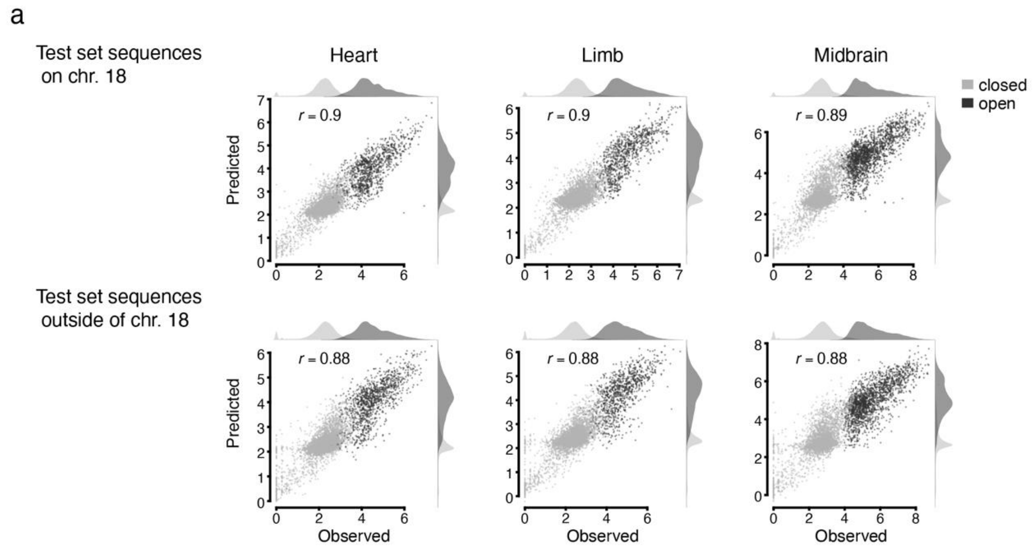


***Supplementary Information:*** *a- Observed vs. predicted ATAC-Seq signal on sequences that were not used for training (test set) and are either found on chromosome 18 (and were excluded from all folds) or on any other chromosome (fold-specific test set that was randomly sampled on all chromosomes).*

2- Extended Data Figure 1a: I am not sure I understand what the goal is to show the % of ATAC peaks overlapping TSS. Are those peaks discarded of the analysis to make sure the model learn with enhancers and not promoters? Could the author please clarify this?

We thank the reviewer for this question. The analysis of TSS overlap was performed to characterize the composition of the peak sets and to assess whether shared ATAC-seq peaks are enriched for promoter-proximal regions, as expected. Indeed, many shared peaks overlap TSSs and likely represent promoters.

These peaks were not discarded during model training or evaluation, and the %-overlap analysis merely provides context for Fig. 1c, where we show that the sequence-to-accessibility models perform well on both tissue-specific (largely TSS-distal) peaks and shared peaks (often TSS-proximal, promoter-associated). We have clarified this in the revised manuscript (lines 73-77).

3- Figure 1b: Could the authors add a scale for the observed ATAC peaks? It would also be informative to have information about what enhancers/gene loci we are looking at.

We added the scale and the genomic coordinates (legend) for the ATAC peaks, and – where available – the proximal genes. Many thanks.

4- Figure 1c: are those regions all from chr18? Could this be stated somewhere?
The regions are not restricted to chromosome 18 but correspond to the held-out test set drawn from all chromosomes and excluded during training (see response to comment #1). We now clarify this in the figure caption.

5- The model rejected 300,000 randomly generated sequences (line 97). How were those sequences generated? Randomly generated sequences can carry TFBS and there are examples in the literature of random sequences driving expression (work from the Crocker lab, for example). Could the author briefly comment on why all the 300,000 randomly generated sequences are rejected by their model?

We thank the reviewer for this question. The randomly generated sequences were constructed by sampling nucleotides uniformly, without enforcing biological constraints. As expected, such sequences can occasionally contain TF binding sites and may therefore receive non-zero scores from the model.

The purpose of this analysis was to assess model specificity. While some random sequences containing one or two relevant motifs show measurable increases in predicted activity (new Extended Data Fig. 2h-i) and a subset even reaches intermediate scores (e.g., around the 0.5 max. likelihood threshold; new Extended Data Fig. 2j, none of the 300,000 sequences passes the stringent activity threshold used in this study:

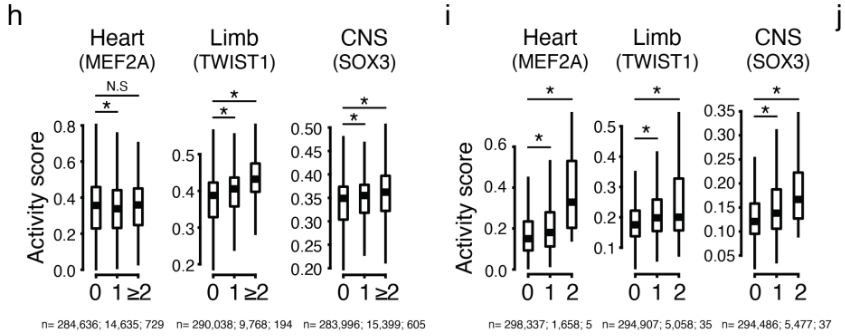


j

Closed genomic regions

| Cutoff | Heart | Limb | CNS |
|---|---|---|---|
| 0.5 | 15.95% | 2.33% | 1.50% |
| 0.8 | 0.00% | 0.00% | 0.00% |

Random sequences

| Cutoff | Heart | Limb | CNS |
|---|---|---|---|
| 0.5 | 2.06% | 0.09% | 0.11% |
| 0.8 | 0.00% | 0.00% | 0.00% |

***Extended Data Fig. 2h-j:*** *h-i- Activity scores of 300,000 closed genomic regions (g) and 300,000 random sequences (h), stratified by the number of tissue-specific motifs they contain (top label). j- Percentage of closed genomic regions and random sequences reaching increasing predicted activity thresholds.*

We have clarified these points in the revised manuscript (lines 102-104.

6- Extended Data Figure1i-j: Can the authors comment on why the model trained directly on VISTA performs so well on the heart compared to other tissues? Also, in j, why is the model trained directly on VISTA not plotted for the midbrain? From i, it seems that it is close to 0 for limb but not for midbrain.

We thank the reviewer for this question. While we do not have a definitive explanation, one biologically plausible interpretation is that the heart may be driven by a more constrained and well-defined set of TFs, similar to our previous observations in Drosophila muscle (de Almeida et al., 2024), where a similarly focused TF repertoire appeared to facilitate learning from limited training data. The heart VISTA subset may therefore present a more learnable regulatory grammar even without the pre-training step. We note, however, that this remains a post-hoc interpretation rather than a tested hypothesis. Regardless of the underlying cause, the transfer-learning approach consistently outperforms direct VISTA training across all three tissues (Extended Data Figure 2k-l, lines 104-109 and 153-171).

Regarding Extended Data Figure 1j (now Extended Data Fig. 2l), we apologize for the lack of clarity. When using random initialization for limb or midbrain, no sequence reaches the activity threshold defined in Extended Data Fig. 2g (previously Extended Data Fig. 1h), and therefore the red bars are at zero for both, limb and midbrain. We have clarified this in the revised figure legend.

7- Figure 2a: could the authors provide the list of all TF motif contributions (either on the figure or better as a supplementary file)?

We now added the names of all TF motifs to Figure 2a and also provide them as a new Supplementary Table 1, including name, PWM, and all scores. Many thanks.

8- Could the authors provide more information on the results of the de novo design of synthetic enhancers using their model? How many sequences per tissue? What was the threshold for scoring these de novo enhancers as good candidates? How were the 5 tested tissue-specific enhancers chosen in this list? Were they the ones with the highest score? It is impressive that all 15 tested enhancers lead to somewhat specific LacZ expression, but I wonder if this is because the author chose the 5 "best" ones. From Supplementary Table 1, it seems that the predicted tissue-

specific accessibility is similar for all synthetic enhancers from the same tissues. Do all predicted enhancers have a similar score?

We apologize for the lack of clarity. Briefly, for each tissue, we generated 1,200 candidate sequences, which received similarly high scores. These candidates were then filtered using thresholds requiring high predicted activity in the target tissue and low predicted activity in other tissues (Extended Data Fig. 3 a-b):

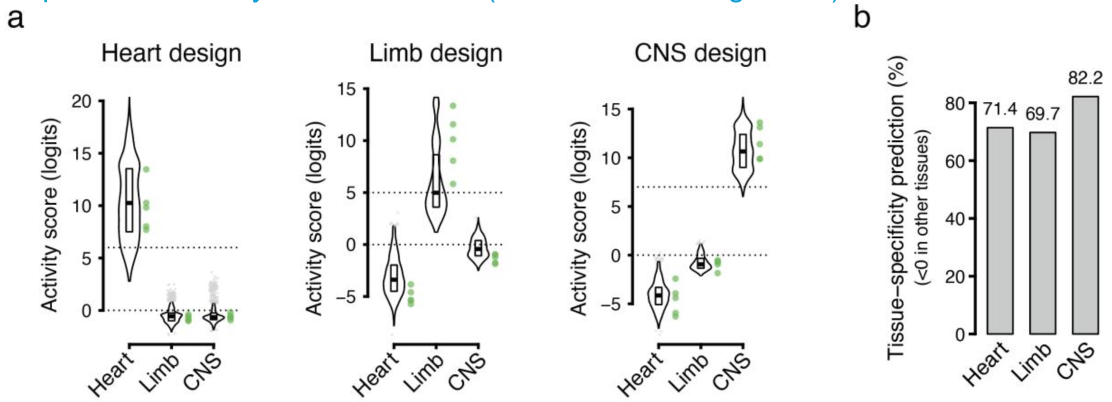


***Extended Data Fig. 3a-b:*** *a- Predicted activity scores (logits) for synthetic enhancer sequences designed for heart (left), limb (center), or CNS (right), using the three sequence-to-activity models (x-axes). The dotted lines indicate the cutoffs used to define sequences predicted to be strongly active in the target tissue (>6, >5, and >7 for heart, limb, and midbrain, respectively) and inactive in the two other tissues (<0). Sequences chosen for validation are shown as green dots. b- Percentage of sequences that are predicted to be strongly active in their target tissue (see previous panel) and inactive (<0) in the two other tissues.*

From this filtered pool, five enhancers per tissue were selected for experimental validation. Importantly, these were not chosen based on the highest predicted scores but were selected to represent diverse motif compositions among the highly-scoring candidate sequences. We now show the individual predicted scores of the validated enhancers (see green dots in panel a right above), demonstrating that they span a representative range of high-scoring candidates.

This selection strategy explains both the comparable predicted activities across enhancers within each tissue and the observed diversity in motif composition and expression patterns (see also point 12 below). We have clarified these points in the revised manuscript.

9- LacZ staining for testing of the synthetic enhancer.
- The methods for the LacZ staining are missing from the Material/Methods section. Particularly, it would be important to know how long the embryos have been stained for (less than 1h, a couple of h or more than 24h?). The better an enhancer works, the quicker the LacZ staining should appear. Were all 15 enhancers stained for the same time?
- Also, how was this compared to a strong endogenous enhancer (for example, the tested VISTA enhancers)? LacZ staining is not a quantifiable assay, but this can be done by RT-qPCR. If possible, could the expression of LacZ be quantitatively compared to an endogenous enhancer?

We thank the reviewer for these questions. We have now added a detailed description of the LacZ staining procedure to the Methods section (lines 557-565). All embryos for all 15 enhancers were processed under identical conditions and

incubated for 24 hours in X-gal staining solution, consistent with standard protocols used for VISTA enhancers.

We note that in 13 out of 15 cases, clear staining was already detectable after approximately 1 hour of incubation, indicating robust enhancer activity. Based on our experience with VISTA enhancers at the H11 locus, such rapid staining is typically observed only for strong enhancers.

However, we did not include a quantitative comparison (e.g., RT-qPCR) or make claims regarding enhancer strength, as LacZ staining is used here as a qualitative readout of enhancer activity and tissue specificity. In addition, the models are trained on the VISTA dataset, which provides categorical annotations rather than quantitative measurements of enhancer strength, and therefore do not explicitly model activity levels.

Quantitative measurements would require entirely new in vivo experiments in mouse embryos, as the current samples are fixed and not amenable to RT-qPCR or similar approaches. Accordingly, we focus on qualitative assessment, consistent with the VISTA framework, and discuss the prediction of enhancer strength in vivo as an attractive direction for future work (lines 238-241). Many thanks.

- On the extended Data Figure, “single” and “tandem” appear. Could the author clarify why certain insertions are in tandem?

We thank the reviewer for this question. We used our previously published site-directed enSERT method to test enhancer activity (Kvon et al., Cell 2020), which employs Cas9 and homology-directed repair to insert a transgene at a specific location (H11 locus) on chromosome 11. This approach results in approximately 50% single-copy insertions and 50% multicopy insertions, typically consisting of 2 – 4 copies at the H11 locus. We have clarified this in the Methods section (lines 568-570) and in the caption of the corresponding Extended Data Fig. 5 (previously Extended Data Fig. 3).

- The embryo shown in the main figure for the heart enhancer #5 is not an E11.5. Could the author use image #2 (from the Extended Data Figure) in the main figure instead? Also, this enhancer #5 clearly shows lower expression than the four others. This should be mentioned.

We thank the reviewer for this observation. We agree that the embryo shown for heart enhancer #5 in the main figure (embryo #1) appears to correspond to an earlier developmental stage (~E10.5). We have therefore replaced it with embryo #2 from the Extended Data Figure in the revised main figure.

We also agree that enhancer #5 shows weaker activity compared to the other heart enhancers and now explicitly note this in the revised manuscript (line 147).

- The embryos for the CNS enhancer #4 are not all looking good. Any specific reason for that?

We thank the reviewer for this observation. The suboptimal morphology of these embryos is due to technical issues during dissection and staining, likely caused by partial evaporation of the staining solution. While this affected overall morphology, robust pan-CNS staining is visible in all six embryos. We have clarified this in the revised figure legend.

- The authors called 9 embryos out of 14 positively stained as heart-specific expression for heart enhancer #1. However, I am not convinced about embryos 8 and 9. The expression is ubiquitous, so I would suggest calling only 7/14 positive embryos.

We thank the reviewer for this careful assessment. We agree that embryos 8 and 9 show more diffuse staining and have therefore revised the scoring to 7/14 embryos with clear heart-specific expression. This has been updated in the figure and corresponding text.

10- Would it be possible to show the functionality of the synthetic enhancer in an endogenous context? For example, knock in one of the synthetic limb enhancers at the Shh locus in a ZRS mutant (maybe add REX?). Would this rescue any expression of Shh? In my opinion, this will be very relevant, and I would love to see something like that, but I am aware that such in vivo experiments can be time-consuming.

We agree that testing synthetic enhancers in an endogenous genomic context would be an interesting extension of this work. The primary goal of the present study, however, is to establish that de novo-designed enhancers can drive tissue-specific activity in vivo in mammalian embryos, using reporter assays directly comparable to the VISTA framework, which represents the current gold standard in the field.

As the reviewer notes, testing enhancer function at endogenous loci introduces additional layers of complexity, including the potential requirement for auxiliary regulatory elements such as range extenders (REX). Such experiments would require substantial additional time and resources and are therefore, in our view, beyond the scope of the present study.

We agree that this is an interesting direction and highlight it as an important avenue for future work in the Discussion (lines 228-236). We also clarify in the revised manuscript that our conclusions are based on reporter assays, and that endogenous testing represents a distinct and more complex setting.

11- Many sequences defined as “enhancers” with the sequence to accessibility model were not shown as enhancers with the VISTA sequence-to-activity model. So, what differentiates the accessible region that can drive expression to the one which cannot? Is it only TFBS? I am not asking for any further experiment here, but maybe some discussion about that.

We thank the reviewer for this insightful question. To address this point, we performed a comparative analysis of genomic regions predicted to be accessible and active versus those predicted to be accessible but inactive.

We find that accessible but inactive regions are more frequently associated with promoters and CTCF-bound sites, and they tend to be accessible across multiple tissues. In contrast, regions that are both accessible and active are more often distal from promoters and CTCF-bound sites and exhibit tissue-specific accessibility (see new Extended Data Fig. d):

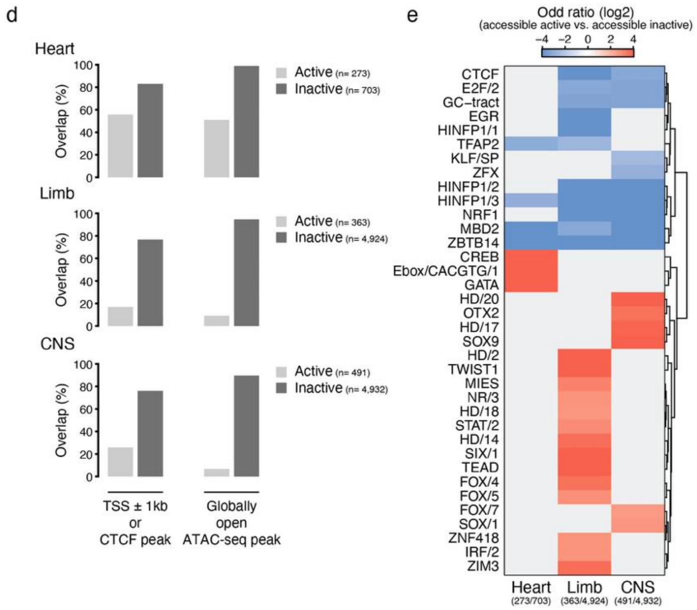


***Extended Data Fig. 3d-e:*** *d- Percentage of overlap between sequences that were predicted to be accessible and active (light grey) or accessible but inactive (dark grey) and annotated promoters (TSS±1kb) or CTCF peaks (left bar) or globally open ATAC-seq peaks (i.e. peaks that are accessible in all three tissues; right). e- Hierarchical clustering of motifs (Euclidean distances) that were significantly over-represented in sequences that were predicted to be accessible and active versus accessible but inactive.*

In addition, the two classes differ in their motif composition (see new Extended Data Fig. 3e panel right above). Accessible but inactive regions are enriched for motifs associated with architectural or broadly active factors (e.g., CTCF), whereas accessible and active regions show enrichment for tissue-specific transcription factor motifs. These differences are consistent with the motif analyses presented in Fig. 2a.

We now include these analysis and discussion in the revised manuscript (lines 122-126).

12- How similar in sequence are the 5 designed enhancers for a specific tissue? From Fig. 2b, it seems that all enhancers have TFBS motifs for the mentioned TF, but what could drive the differences in the LacZ expression pattern? Do they all have the same number and order of TFBS and similar spacing between those motifs?

We thank the reviewer for this question. While the designed enhancers for a given tissue share key transcription factor motifs, they are not identical in sequence composition (please see new Extended Data Fig. 4d and f, shown above in our response to reviewer 1, point 1). Importantly, the five enhancers per tissue were selected from a pool of high-scoring candidates to represent diversity in motif content rather than maximal predicted activity (please see our response to point 8 above).

As a result, the enhancers differ in the presence or absence of specific motifs, as well as in the number of motif instances. These differences are now illustrated in the new Extended Data Fig. 4c, where we compare motif composition across the designed enhancers:

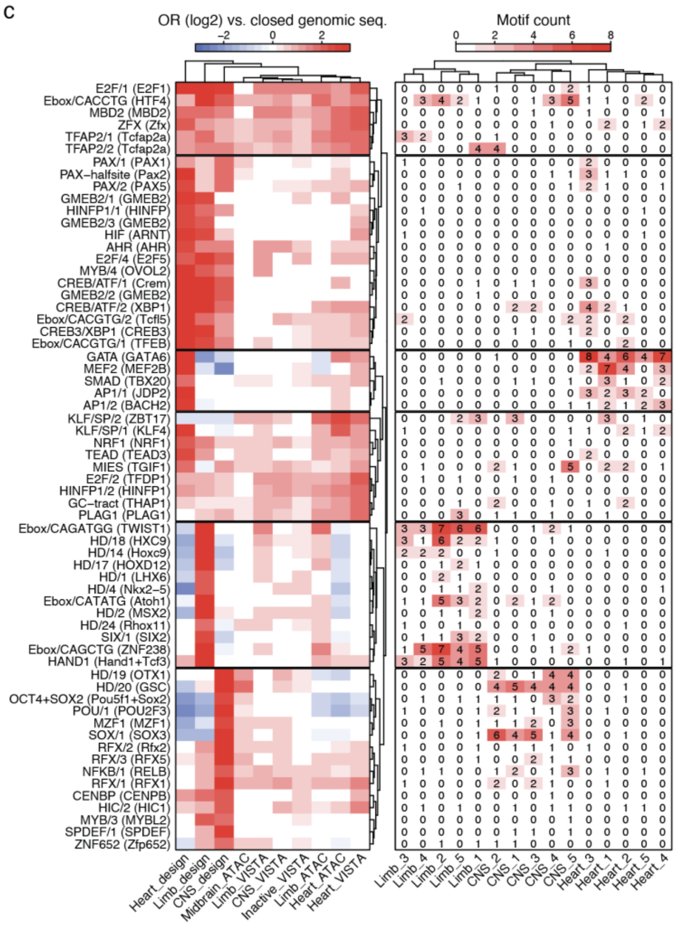


***Extended Data Fig. 4c:*** *Left: hierarchical clustering of the top significantly over-represented motifs in designed enhancer sequences, inactive or active VISTA sequences, and ATAC-seq peaks accessible in each of the three target tissues (see x axis labels). Right: corresponding motif counts within the 15 validated synthetic enhancers (see x axis labels).*

Such variation in motif content and organization is consistent with the observed differences in LacZ expression patterns, suggesting that distinct combinations and configurations of transcription factor binding sites can give rise to related but non-identical activity patterns.

Minor comments:
1- Since the submission of this paper, a new work was published using MPRA-based deep learning models to design synthetic human promoter sequences (https://doi.org/10.1038/s41586-025-10093-z). Citing this work might be relevant.

We agree and now cite the work (ref #12), many thanks.

2- Please define the acronym PCC (Pearson Correlation Coefficient) in the main text. It currently appears only in the methods.

Fixed, many thanks.

3- Extended data 1b is cited before 1a in the text.
Fixed, many thanks.

**Reviewer #3 (Remarks to the Author):**
Chen et al. present an approach to design mammalian tissue-specific enhancer sequences using a deep learning model. The approach first trains a relatively simple CNN to predict ATAC-seq signals across three tissue types (heart, limb, midbrain). The model is then fine-tuned using a small set of experimentally validated enhancer sequences from the VISTA enhancer browser. The Ledidi model is then used to generate synthetic enhancer sequences from the trained CNN and several generated sequences were tested for enhancer activity in mouse embryos. While other studies have demonstrated the feasibility of using deep learning models to generate synthetic enhancer sequences, the importance of this study lies in the simple training procedure (i.e., relying first on an ATAC-seq training objective). Previous studies have relied on MPRA training data, so the feasibility of training with accessibility data greatly broadens the number of cell/tissue types that might be targeted by enhancer design techniques. This is also, to my knowledge, the first study that characterizes enhancer activity of designed sequences in mouse embryos, demonstrating the success of the approach.

We thank the reviewer for the positive evaluation of our work and for highlighting the simplicity of the training strategy and the use of accessibility data as key advances that broaden the applicability of enhancer design approaches. We are also grateful to the reviewer for recognizing this as the first study demonstrating the activity of designed enhancers in mouse embryos. We appreciate the reviewer’s constructive suggestions for clarification and address both points below.

Overall, this is a compact and well-executed study. I only have two minor comments for clarification:
1. The methods state that "seed sequences were then optimized using both the sequence-to-accessibility and sequence-to-activity models...". This should be clarified. Were distinct sequences designed using each model? If so, which models generated each of the tested sequences? Or, alternatively, was there some form of joint optimization using both models?

We thank the reviewer for this question. The designed sequences were generated by jointly optimizing both the sequence-to-accessibility and sequence-to-activity models. Specifically, we used Ledidi to introduce edits into random starting sequences to achieve predefined target values for both models simultaneously (see Methods and Extended Data Fig. 4a-b).

Thus, a single sequence is optimized to satisfy both accessibility and activity objectives, rather than generating separate sequences for each model. We have clarified this in the Methods section (lines 490-505).

2. Figure 2a shows how motif contribution scores change before and after transfer learning. It would be more informative to label all rows in this figure.
We thank the reviewer for pointing out that labeling all rows would be informative and improves interpretability. We therefore labelled all rows and further provided a new Supplementary Table 1 containing the IDs, names and PWMs of all the motifs, together with the associated mean contribution values.

**Reviewer #1 (Remarks to the Author):**

The authors addressed the concern of similarity of the designed enhancers to VISTA sequences adequately. The authors also added additional analyses using chromatin features for fine-tuning in place of VISTA, which substantially widens the applicability of their approach to other biological systems. The authors also added analyses of potential novel and higher-order motif patterns which may inform future functional studies beyond the scope of this manuscript. Altogether, the authors have adequately addressed the concerns I raised during first round of review. In my view, this manuscript advances the field of genetics in a substantial manner and is suitable for publication.

Reviewer #1 (Remarks on code availability):

Please ensure that the code that uses chromatin features as a replacement for VISTA for fine-tuning are also shared.

All our code, including the code that uses chromatin features as a replacement for VISTA for fine-tuning, is shared on GitHub as we now clarified in the revised "Code availability" section.

**Reviewer #2 (Remarks to the Author):**

The authors have answered most of my questions. I am overall satisfied by their answers and additional analysis and data. However, before publication, I am asking for some minor corrections:

1) On Figure 2b, #enhancer 1 is still indicated as having 9/14 embryos with similar staining while I asked the reviewers to correct that. See previous comment point 9 and their answer here:
"- The authors called 9 embryos out of 14 positively stained as heart-specific expression for heart enhancer #1. However, I am not convinced about embryos 8 and 9. The expression is ubiquitous, so I would suggest calling only 7/14 positive embryos.
We thank the reviewer for this careful assessment. We agree that embryos 8 and 9 show more diffuse staining and have therefore revised the scoring to 7/14 embryos with clear heart-specific expression. This has been updated in the figure and corresponding text."

We are sorry for the oversight when putting together the main figures for resubmission – the Extended Data Figure 5 already had the updated numbers. This is now fixed for the main figure 2 as well, many thanks.

2) The authors have added Figure 3 showing the validation of forebrain and midbrain-specific enhancers. In the current revised version of the manuscript, the text describing this figure is in the Discussion part. This should be move to the results. As indicated by

the authors in the manuscript ("Together, these results demonstrate that ..." line 224-225), this is a result and not a discussion point.

We agree that the experiments shown in Figure 3 represent results, now for two designed forebrain enhancers. We had placed these data in the Discussion because we view them primarily as an outlook on future extensions of the framework rather than as a central result of the present study. This original decision had been based on the main focus of this work being the design of tissue-specific enhancers at the level of major embryonic tissues (heart, limb, and CNS), for which we provide 15/15 validated synthetic enhancers. By contrast, the forebrain/midbrain enhancers represent an exploratory proof-of-principle rather than a fully developed component of the study, and are therefore best discussed as part of the outlook.

However, we agree with the reviewer that Figure 3 represents novel results and followed the request to present these in the Results, thanks.

3) This new Figure 3 shows embryos with GFP and/ or mCherry expression. When testing MB1 and FB1, the authors are showing only one embryo and indicate that this staining was observed for 1 out of 1 embryo. These are not statistically relevant results, and I would like to see replicates for that construct and at least 3 embryos showing similar stainings. It is particularly important as we can observe from the current Extended data 8c that the staining is highly variable between different embryos (for FB2). I believe that this needs to be corrected before publication.

We agree that a single embryo is insufficient to assess reproducibility, particularly given the variability observed among the forebrain enhancer constructs. We therefore performed two additional rounds of transgenic injections.

These experiments provided additional support for the forebrain enhancer FB1, bringing the total to 8/9 embryos with consistent forebrain activity (FB2 already had 4/5 embryos and was not retested). For the midbrain enhancer MB1, we obtained one additional embryo with midbrain activity, whereas seven other embryos showed no reproducible reporter expression.

However, full-plasmid sequencing and restriction digestion analysis revealed a 419-bp duplication in one of the two midbrain transgene insertions, precluding interpretation of the result. This duplication was missed in the original QC analysis because the plasmidsaurus whole-plasmid-sequencing software failed to correctly assemble the repeated regions. We have now double-checked the sequences and digestion patterns for all LacZ and fluorescent reporter constructs and confirmed their accuracy. We updated the text, Figure 3, Extended Data Figure 8, and the discussion to reflect the new results on the forebrain and midbrain enhancers.

The revised manuscript therefore presents two validated forebrain enhancers (8/9 and 4/5 embryos) and one midbrain enhancer that did not show reproducible activity (0/5 embryos). The duplicated MB1 construct is reported only for completeness and is not interpreted.

**Reviewer #3 (Remarks to the Author):**

I had only minor comments on the first manuscript submission. The revised manuscript is even further improved. I have no further comments at this time.

Many thanks.
